# Simultaneous Ablation of Neuronal Neurofascin and Ankyrin G in Young and Adult Mice Reveals Age-Dependent Increase in Nodal Stability in Myelinated Axons and Differential Effects on the Lifespan

**DOI:** 10.1523/ENEURO.0138-18.2018

**Published:** 2018-06-27

**Authors:** Anna M. Taylor, Qian Shi, Manzoor A. Bhat

**Affiliations:** Department of Cellular and Integrative Physiology, Center for Biomedical Neuroscience, University of Texas Health Science Center, San Antonio, Tx 78229

**Keywords:** Ankyrin G, Axon degeneration, maintenance of nodes, myelination, nodes of Ranvier

## Abstract

Nodes of Ranvier are unique regions where voltage-gated sodium channels are highly enriched to drive saltatory conduction. Genetic ablations in adult mice with loss of specific nodal proteins causes slow but progressive nodal deterioration associated with decreased nerve conduction and axonopathy. What has remained unaddressed is whether loss of nodal proteins at different time points in postnatal life follows similar timelines of nodal disorganization. Here we utilized simultaneous ablation of Neurofascin (NF186) and Ankyrin G (AnkG) in mice of both sexes at three specific time points. We report that concurrent ablation of these core nodal components at postnatal day 13 (P13) leads to accelerated nodal destabilization in comparison with P23, and this disorganization is even slower when ablated at P93. Ablation of NF186 with AnkG at P13 reduced the half-life of NF186 to 15 days compared to 1 month at P23, which increased to 2 months at P93, indicating increasing nodal stability. The half-life of AnkG at the nodes also increased with age but showed enhanced disappearance from the node in the absence of NF186, with a half-life of 3 days at P13 ablation. The nodal disorganization occurred in a sequential manner, with AnkG disappearing first from the nodal areas irrespective of the timing of ablation, and led to decreased nerve conduction and affected axonal health. Together, our studies reveal that nodes of Ranvier in myelinated axons continue to become more stable with age and suggest that nodal disorganization in adult human demyelinating disorders occurs slowly until neurological symptoms become evident.

## Visual Abstract

**Figure:**
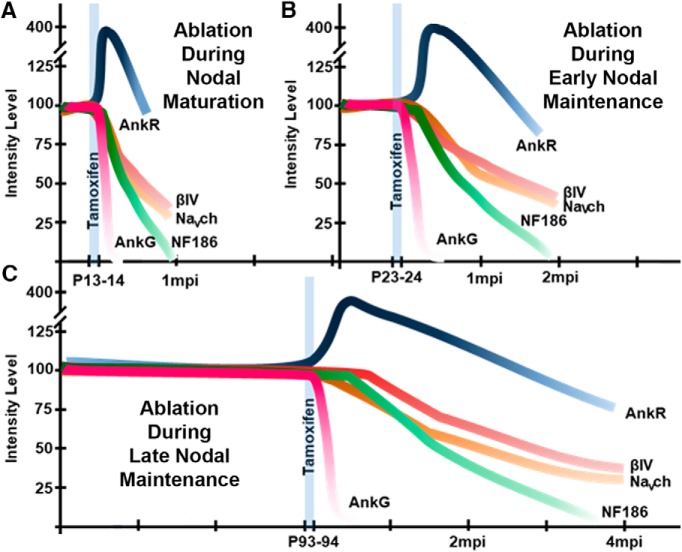

## Significance Statement

The initial clustering, maturation, and lifelong maintenance of nodes of Ranvier along myelinated axons are vital to the saltatory propagation of action potentials, allowing for rapid movements. To investigate if the kinetics for nodal destabilization are variable with age, we used a novel mouse model in which two essential nodal proteins, Neurofascin 186 (NF186) and Ankyrin G (AnkG), were simultaneous ablated from neurons at different time points. Our studies show not only that nodal stability after maturation is dependent on the synergistic functions of these core nodal components, but also that nodes become more stable as animals age, which is relevant to human demyelination disorders.

## Introduction

Intricate molecular complexes evolved to cluster ion channels into distinct domains along axons, including nodes of Ranvier (nodes), to allow for rapid movements in higher-order mammals. The nodal complex, which is critical to the saltatory propagation of action potentials, requires cell adhesion molecules as well as cytoskeletal scaffolding components to anchor ion channels. The core components of nodes have been well defined to include the 186-kDa neuron-specific isoform of Neurofascin (*Nfasc^NF186^*), NrCAM, Ankyrin-G (AnkG), βIV Spectrin, and voltage-gated sodium (Na_V_) channels (Kordeli et al., 1995; Davis et al., 1996; Lambert et al., 1997; Komada and Soriano, 2002). However, the precise order in which these nodal components arrive and their individual roles in the establishment, maturation, and maintenance of nodes remain heavily debated (Buttermore et al., 2013; Nelson and Jenkins, 2017).

Recent focus, which has been fueled by the use of spatio-temporal ablation as well as double knockout of nodal components, has been to elucidate how the node is maintained throughout the lifespan and how this ultimately relates to the health of myelinated axons (Pillai et al., 2009; Zonta et al., 2011; Zhang et al., 2012; Amor et al., 2014; Desmazieres et al., 2014; Saifetiarova et al., 2017; Taylor et al., 2017). Specifically through the use of an inducible, neuronal-specific driver to ablate either AnkG or NF186 from mature nodes, we previously reported that while these two key proteins are both necessary for the long-term maintenance of the node, they have inherently different stabilities, leading to differential kinetics of nodal disorganization and axonal pathology (Saifetiarova et al., 2017; Taylor et al., 2017). In both cases, disrupting the nodal complex after it was fully formed took more than half a year; however, ablating AnkG using this same model at an early time point before nodal formation resulted in nodes that clustered but then began to deteriorate (Saifetiarova et al., 2017). Instead of a pioneering role in the initial formation of the node, as was previously proposed (Jenkins and Bennett, 2002; Dzhashiashvili et al., 2007; Jenkins et al., 2015), these studies reveal an essential functional for AnkG in the stabilization of the node into a mature complex. Furthermore, these studies suggested that cytoskeletal and cell adhesion components play unique roles in nodal stability during specific stages of postnatal life.

In the current study, we addressed the combined roles of a cytoskeletal scaffolding component and a transmembrane cell adhesion protein in nodal stabilization, as well as the impact of age on nodal stability, by taking advantage of spatio-temporal ablation of both AnkG and NF186 at multiple time points from early maturation to long-term maintenance. We show that simultaneous ablation of AnkG along with NF186 from a fully formed node further reduces the stability of NF186, leading to enhanced kinetics of nodal destabilization, nerve conduction loss, and axonopathy compared to the single-ablation models. Inducing ablation of AnkG in combination with NF186 at an earlier time point led to even more rapid decline of NF186 stability and consequently the levels of Na_V_ channels at the node. Although delaying ablation to later stages slowed nodal deterioration, loss of AnkG and NF186 ultimately had similar detrimental consequences on axonal health. Together, our studies suggest that nodal maturation occurs over time such that nodal complexes in older mice destabilize much more slowly than in younger mice, and that this age-dependent maturation increases the half-life of nodal proteins to ensure an optimal rate of nerve conduction by myelinated axons.

## Materials and Methods

### Materials

All chemical and reagents were purchased from Sigma-Aldrich, unless otherwise specified. Antisera used, which were previously described, include rabbit anti-AnkG, anti–βIV Spectrin, and anti–pan Na_V_ Channels (Taylor et al., 2017), guinea pig and rabbit anti-Caspr (Bhat et al., 2001), guinea pig anti-NF186 (Thaxton et al., 2011), and rat anti-NFCT (Pillai et al., 2009). Primary antibodies include rabbit anti-Gapdh (RRID:AB_796208, #G9545); mouse anti–β-actin (RRID:AB_476744, #A-5441); mouse anti-Caspr (RRID:AB_2083496, #75-001, NeuroMab); anti-AnkR (RRID:AB_2491109, #75-380, NeuroMab); and anti-Kv1.2 (RRID:AB_2296313, #75-008, NeuroMab). Fluorescent secondary antibodies (Alexa Fluor; Life Technologies) and infrared (IR)-conjugated secondary antibodies (LI-COR) were also purchased. Reagents used to perform electron microscopy were from Electron Microscopy Sciences.

### Animals and treatments

To characterize the combined role of AnkG and Neurofascin in nodal maturation and maintenance, *Ankyrin3^Flox^* (*Ank^fx^*) mice (RRID:MGI:5538267), which were provided by Vann Bennett (Duke University; Paez-Gonzalez et al., 2011), and *Nfasc^Flox^* (*NF^fx^*) mice (Pillai et al., 2009) were bred to generate a double *NF^fx^;Ank^fx^* mouse line. This double floxed line was then crossed with the single-neuron labeling with inducible Cre-mediated knockout (SLICK)-H (derivative of *Thy1.2-CreER*) transgenic mouse line (RRID:IMSR_JAX:012708), which were provided by Paul Young (Heimer-McGinn and Young, 2011), to specifically knock out AnkG and NF186 in neurons at various time points. Then, *SLICK-H;NF^+/fx^;Ank^+/fx^* were bred to *NF^+/fx^*;*Ank^+/fx^* to generate controls (*NF^fx/fx^;Ank^fx/fx^;*), single AnkG conditional knockouts (cKOs; *SLICK-H;NF^+/+^;Ank^fx/fx^*), single NF186 cKOs (*SLICK-H;NF^fx/fx^;Ank^+/x^*), and double AnkG/NF186 cKOs (*SLICK-H;NF^fx/fx^;Ank^fx/fx^*). These mice were maintained on a mixed-strain background of C57BL/6 and 129/Vs. Mice were genotyped before postnatal day 12 (P12) and weaned by P21. The mice were group-housed in a temperature-controlled animal facility (23 ± 1°C) with a maintained light cycle (12 h light on/12 h off) and *ad libitum* access to water and a standard rodent diet.

To induce genetic ablation of AnkG, NF186, or both, tamoxifen (MP Biomedicals) suspended in sunflower seed oil (1 mg/µL) was delivered as intraperitoneal (i.p.) injections for 2, 5, or 10 consecutive days. As no increase in recombination was seen with additional doses, all studies presented in this manuscript used 2 consecutive doses of tamoxifen, either from P13/14, P23/24, or P93/94. To determine the dose of tamoxifen given, each mouse was weighed at the time of injection and injected with 1 mg/12.5 g body weight, which was the average weight calculated for P23/24 *NF^fx^;Ank^fx^* mice and thus the dose used for previous published single AnkG and NF186 studies when 1 mg was given daily between P23 and P32 (Saifetiarova et al., 2017; Taylor et al., 2017). At various time points postinjection, age-matched control, single AnkG cKO, and double AnkG/NF186 cKO littermates were evaluated by electrophysiological, immunohistochemical, biochemical, and ultrastructural techniques.

To promote survival after tamoxifen ablation of NF186 and AnkG, all mice regardless of genotype were provided with moist food on the floor of their cage after weaning. End stage was defined as the time at which mice placed on their backs could no longer right themselves. At this terminal stage, mice were humanely euthanized. All animal research was performed with prior approval from the Institutional Animal Care and Use Committee of the University of Texas Health Science Center at San Antonio in accordance with the Public Health Service Policy on Humane Care and Use of Laboratory Animals.

### Experimental design and statistical analysis

For each experiment described, the number of mice used was designed based on the minimal number of animals necessary to determine high statistical differences (P < 0.01) between genotypes in previously reported studies. As no gender differences have been revealed at nodes of Ranvier, we used equal numbers of males and females per group (each genotype at all three different ablation timelines). For survival studies, 8–10 mice were used per group, and 6–8 mice per group at each time point postinjection studied were used for nerve conduction velocity (NCV) recordings. For immunostaining, tissues were processed from 3–4 mice per group per time point, then 50–70 nodes from PNS and 80–100 nodes from CNS were quantified per animal. For immunoblotting, tissues were processed and quantified from 3 mice per group at the terminal time point. For ultrastructure analyses, tissues were processed from 3 mice per group, and for each mouse a minimum of 200 axons for SN and 500 axons for SC were imaged.

All data are presented as the mean ± SEM, where the *n* always equals the number of mice/group. Relative values were arithmetically adjusted to yield a unit of 1 for the control group at each time point. To compare multiple time points across genotypes, two-way ANOVAs were performed followed by Tukey’s multiple comparison analyses. When only a single time point was used, statistically significant differences between genotypes were determined using unpaired, two-tailed Student’s *t* tests. Statistical differences are represented in figures by * (P < 0.05), ** (P < 0.01), *** (P < 0.001) with black asterisks indicating differences between age-matched control and mutants; colored asterisks signify differences among the mutant groups at different ages (green for P13/14 induced, blue for P23/24 induced double, and red for P93/94 induced double AnkG/NF186 cKO). Significant differences in survival curves were determined by Mantel–Cox log-rank test, which were evaluated individually and then corrected for multiple comparisons with the Bonferroni method. All statistical tests were performed using GraphPad Prism6 software (RRID:SCR_002798) and are summarized with exact *p*-values in Table 1.

**Table 1.** Statistical summary

Item	Figure	Data structure	Type of test	Description	Comparison	Power
a	1*F*	Normal distribution	Mantel–Cox log rank test; Bonferroni *post hoc* analysis; *K* = 6	Survival postinjection		
					Control vs. P13 ablation DKO	χ^2^ = 51.18; *p* < 0.0001
					P13 ablation DKO vs. P23 ablation DKO	χ^2^ = 25.28; *p* < 0.0001
					P13 ablation DKO vs. P93 ablation DKO	χ^2^ = 17.72; *p* < 0.0001
					Control vs. P23 ablation DKO	x2 = 31.28; *p* < 0.0001
					P23 ablation vs. P93 ablation	χ^2^ = 18.68; *p* < 0.0001
					Control vs. P93 ablation	χ^2^ = 8.0; *p* = 0.0047
b	1*G*	Normal distribution	Two-way ANOVA; Tukey *post hoc* analysis	P13 ablation: body weight		
					Effect of genotype	*p* < 0.0001
					Effect of timing postinjection	*p* < 0.0001
					Genotype × timing interaction	*p* = 0.4746
					Control 10 dpi vs. DKO 10 dpi	*p* = 0.1806
					Control 20 dpi vs. DKO 20 dpi	*p* = 0.0038
					Control 30 dpi vs. DKO 30 dpi	*p* = 0.0023
c	1*H*	Normal distribution	Two-way ANOVA; Tukey *post hoc* analysis	P23 ablation: body weight		
					Effect of genotype	*p* < 0.0001
					Effect of timing postinjection	*p* = 0.0457
					Genotype × timing interaction	*p* = 0.0002
					Control 10 dpi vs. DKO 10 dpi	*p* = 0.9953
					Control 20 dpi vs. DKO 20 dpi	*p* = 1943
					Control 60 dpi vs. DKO 60 dpi	*p* < 0.0001
d	1*I*	Normal distribution	Two-way ANOVA; Tukey *post hoc* analysis	P93 ablation: body weight		
					Effect of genotype	*p* = 1410
					Effect of timing postinjection	*p* < 0.0001
					Genotype × timing interaction	*p* = 0.2562
					Control 10 dpi vs. DKO 10 dpi	*p* > 0.9999
					Control 20 dpi vs. DKO 20 dpi	*p* = 0.9996
					Control 1200 dpi vs. DKO 120 dpi	*p* = 0.3155
e	2*J*, AnkG	Normal distribution	Two-way ANOVA; Tukey *post hoc* analysis	SN P13 ablation: AnkG nodal count		
					Effect of genotype	*p* < 0.0001
					Effect of timing postinjection	*p* < 0.0001
					Genotype × timing interaction	*p* < 0.0001
					Control 10 dpi vs. Ank cKO 10 dpi	*p* = 0.0694
					Control 20 dpi vs. Ank cKO 20 dpi	*p* < 0.0001
					Control 30 dpi vs. Ank cKO 30 dpi	*p* < 0.0001
					Control 10 dpi vs. DKO 10 dpi	*p* < 0.0001
					Control 20 dpi vs. DKO 20 dpi	*p* < 0.0001
					Control 30 dpi vs. DKO 30 dpi	*p* < 0.0001
					Ank cKO 10 dpi vs. 20 dpi	*p* = 0.0010
					Ank cKO 10 dpi vs. 30 dpi	*p* < 0.0001
					Ank cKO 20 dpi vs. 30 dpi	*p* < 0.0001
					DKO 10 dpi vs. 20 dpi	*p* > 0.9999
					DKO 10 dpi vs. 30 dpi	*p* = 0.9997
					DKO 20 dpi vs. 30 dpi	*p* > 0.9999
f	2*J*, NF186	Normal distribution	Two-way ANOVA; Tukey *post hoc* analysis	SN P13 ablation: NF186 nodal count		
					Effect of genotype	*p* < 0.0001
					Effect of timing postinjection	*p* < 0.0001
					Genotype × timing interaction	*p* < 0.0001
					Control 30 dpi vs. Ank cKO 30 dpi	*p* > 0.9999
					Control 10 dpi vs. DKO 10 dpi	*p* = 0.9984
					Control 20 dpi vs. DKO 20 dpi	*p* = 0.0015
					Control 30 dpi vs. DKO 30 dpi	*p* < 0.0001
					DKO 10 dpi vs. 20 dpi	*p* = 0.0042
					DKO 10 dpi vs. 30 dpi	*p* < 0.0001
					DKO 20 dpi vs. 30 dpi	*p* < 0.0001
g	2*J*′, AnkG	Normal distribution	Two-way ANOVA; Tukey *post hoc* analysis	SC P13 ablation: AnkG nodal count		
					Effect of genotype	*p* < 0.0001
					Effect of timing postinjection	*p* < 0.0001
					Genotype × timing interaction	*p* < 0.0001
					Control 10 dpi vs. Ank cKO 10 dpi	*p* < 0.0001
					Control 20 dpi vs. Ank cKO 20 dpi	*p* < 0.0001
					Control 30 dpi vs. Ank cKO 30 dpi	*p* < 0.0001
					Control 10 dpi vs. DKO 10 dpi	*p* < 0.0001
					Control 20 dpi vs. DKO 20 dpi	*p* < 0.0001
					Control 30 dpi vs. DKO 30 dpi	*p* < 0.0001
					Ank cKO 10 dpi vs. 20 dpi	*p* = 0.0011
					Ank cKO 10 dpi vs. 30 dpi	*p* < 0.0001
					Ank cKO 20 dpi vs. 30 dpi	*p* < 0.0001
					DKO 10 dpi vs. 20 dpi	*p* = 0.9973
					DKO 10 dpi vs. 30 dpi	*p* > 0.9998
					DKO 20 dpi vs. 30 dpi	*p* = 0.9996
h	2*J*′, NF186	Normal distribution	Two-way ANOVA; Tukey *post hoc* analysis	SC P13 ablation: NF186 nodal count		
					Effect of genotype	*p* < 0.0001
					Effect of timing postinjection	*p* < 0.0001
					Genotype × timing interaction	*p* < 0.0001
					Control 30 dpi vs. Ank cKO 30 dpi	*p* > 0.9999
					Control 10 dpi vs. DKO 10 dpi	*p* = 0.5624
					Control 20 dpi vs. DKO 20 dpi	*p* < 0.0001
					Control 30 dpi vs. DKO 30 dpi	*p* < 0.0001
					DKO 10 dpi vs. 20 dpi	*p* = 0.0001
					DKO 10 dpi vs. 30 dpi	*p* < 0.0001
					DKO 20 dpi vs. 30 dpi	*p* < 0.0001
i	2*K*, AnkG	Normal distribution	Two-way ANOVA; Tukey *post hoc* analysis	SN P23 ablation: AnkG nodal count		
					Effect of genotype	*p* < 0.0001
					Effect of timing postinjection	*p* < 0.0001
					Genotype × timing interaction	*p* < 0.0001
					Control 10 dpi vs. Ank cKO 10 dpi	*p* = 0.9389
					Control 20 dpi vs. Ank cKO 20 dpi	*p* < 0.0001
					Control 60 dpi vs. Ank cKO 60 dpi	*p* < 0.0001
					Control 10 dpi vs. DKO 10 dpi	*p* < 0.0001
					Control 20 dpi vs. DKO 20 dpi	*p* < 0.0001
					Control 60 dpi vs. DKO 60 dpi	*p* < 0.0001
					Ank cKO 10 dpi vs. 20 dpi	*p* = 0.0008
					Ank cKO 10 dpi vs. 60 dpi	*p* < 0.0001
					Ank cKO 20 dpi vs. 60 dpi	*p* < 0.0001
					DKO 10 dpi vs. 20 dpi	*p* > 0.9999
					DKO 10 dpi vs. 60 dpi	*p* > 0.9999
					DKO 20 dpi vs. 60 dpi	*p* = 0.8804
j	2*K*, NF186	Normal distribution	Two-way ANOVA; Tukey *post hoc* analysis	SN P23 ablation: NF186 nodal count		
					Effect of genotype	*p* < 0.0001
					Effect of timing postinjection	*p* < 0.0001
					Genotype × timing interaction	*p* < 0.0001
					Control 60 dpi vs. Ank cKO 60 dpi	*p* > 0.9999
					Control 10 dpi vs. DKO 10 dpi	*p* = 0.9943
					Control 20 dpi vs. DKO 20 dpi	*p* = 0.0031
					Control 60 dpi vs. DKO 60 dpi	*p* < 0.0001
					DKO 10 dpi vs. 20 dpi	*p* = 0.0089
					DKO 10 dpi vs. 60 dpi	*p* < 0.0001
					DKO 20 dpi vs. 60 dpi	*p* < 0.0001
k	2*K*′, AnkG	Normal distribution	Two-way ANOVA; Tukey *post hoc* analysis	SC P23 ablation: AnkG nodal count		
					Effect of genotype	*p* < 0.0001
					Effect of timing postinjection	*p* < 0.0001
					Genotype × timing interaction	*p* < 0.0001
					Control 10 dpi vs. Ank cKO 10 dpi	*p* < 0.0001
					Control 20 dpi vs. Ank cKO 20 dpi	*p* < 0.0001
					Control 60 dpi vs. Ank cKO 60 dpi	*p* < 0.0001
					Control 10 dpi vs. DKO 10 dpi	*p* < 0.0001
					Control 20 dpi vs. DKO 20 dpi	*p* < 0.0001
					Control 60 dpi vs. DKO 60 dpi	*p* < 0.0001
					Ank cKO 10 dpi vs. 20 dpi	*p* < 0.0001
					Ank cKO 10 dpi vs. 60 dpi	*p* < 0.0001
					Ank cKO 20 dpi vs. 60 dpi	*p* < 0.0001
					DKO 10 dpi vs. 20 dpi	*p* > 0.9999
					DKO 10 dpi vs. 60 dpi	*p* > 0.9999
					DKO 20 dpi vs. 60 dpi	*p* = 0.9991
l	2*K*′, NF186	Normal distribution	Two-way ANOVA; Tukey *post hoc* analysis	SC P23 ablation: NF186 nodal count		
					Effect of genotype	*p* < 0.0001
					Effect of timing postinjection	*p* < 0.0001
					Genotype × timing interaction	*p* < 0.0001
					Control 60 dpi vs. Ank cKO 60 dpi	*p* > 0.9999
					Control 10 dpi vs. DKO 10 dpi	*p* = 0.6324
					Control 20 dpi vs. DKO 20 dpi	*p* = 0.0001
					Control 60 dpi vs. DKO 60 dpi	*p* < 0.0001
					DKO 10 dpi vs. 20 dpi	*p* = 0.0009
					DKO 10 dpi vs. 60 dpi	*p* < 0.0001
					DKO 20 dpi vs. 60 dpi	*p* < 0.0001
m	2*L*, AnkG	Normal distribution	Two-way ANOVA; Tukey *post hoc* analysis	SN P93 ablation: AnkG nodal count		
					Effect of genotype	*p* < 0.0001
					Effect of timing postinjection	*p* = 0.9562
					Genotype × timing interaction	*p* = 0.1124
					Control 10 dpi vs. DKO 10 dpi	*p* < 0.0001
					Control 20 dpi vs. DKO 20 dpi	*p* < 0.0001
					Control 120 dpi vs. DKO 120 dpi	*p* < 0.0001
					DKO 10 dpi vs. 20 dpi	*p* = 0.7683
					DKO 10 dpi vs. 120 dpi	*p* = 0.9502
					DKO 20 dpi vs. 120 dpi	*p* = 0.9925
n	2*L*, NF186	Normal distribution	Two-way ANOVA; Tukey *post hoc* analysis	SN P93 ablation: NF186 nodal count		
					Effect of genotype	*p* < 0.0001
					Effect of timing postinjection	*p* < 0.0001
					Genotype × timing interaction	*p* < 0.0001
					Control 10 dpi vs. DKO 10 dpi	*p* = 0.1372
					Control 20 dpi vs. DKO 20 dpi	*p* = 0.0182
					Control 120 dpi vs. DKO 120 dpi	*p* < 0.0001
					DKO 10 dpi vs. 20 dpi	*p* = 0.9704
					DKO 10 dpi vs. 120 dpi	*p* < 0.0001
					DKO 20 dpi vs. 120 dpi	*p* < 0.0001
o	2*L*′, AnkG	Normal distribution	Two-way ANOVA; Tukey *post hoc* analysis	SC P93 ablation: AnkG nodal count		
					Effect of genotype	*p* < 0.0001
					Effect of timing postinjection	*p* = 0.0083
					Genotype × timing interaction	*p* = 0.0063
					Control 10 dpi vs. DKO 10 dpi	*p* < 0.0001
					Control 20 dpi vs. DKO 20 dpi	*p* < 0.0001
					Control 120 dpi vs. DKO 120 dpi	*p* < 0.0001
					DKO 10 dpi vs. 20 dpi	*p* = 0.1040
					DKO 10 dpi vs. 120 dpi	*p* = 0.0010
					DKO 20 dpi vs. 120 dpi	*p* = 0.2210
*p*	2*L*′, NF186	Normal distribution	Two-way ANOVA; Tukey *post hoc* analysis	SC P93 ablation: NF186 nodal count		
					Effect of genotype	*p* < 0.0001
					Effect of timing postinjection	*p* < 0.0001
					Genotype × timing interaction	*p* < 0.0001
					Control 10 dpi vs. DKO 10 dpi	*p* = 0.9167
					Control 20 dpi vs. DKO 20 dpi	*p* = 0.4298
					Control 120 dpi vs. DKO 120 dpi	*p* < 0.0001
					DKO 10 dpi vs. 20 dpi	*p* = 0.9131
					DKO 10 dpi vs. 120 dpi	*p* < 0.0001
					DKO 20 dpi vs. 120 dpi	*p* < 0.0001
q	2*M*, P13	Normal distribution	Two-way ANOVA; Tukey *post hoc* analysis	SN P13 ablation: NF186 intensity		
					Effect of genotype	*p* < 0.0001
					Effect of timing postinjection	*p* = 0.0001
					Genotype × timing interaction	*p* = 0.0001
					Control 10 dpi vs. DKO 10 dpi	*p* = 0.4702
					Control 20 dpi vs. DKO 20 dpi	*p* = 0.0001
					Control 30 dpi vs. DKO 30 dpi	*p* < 0.0001
					DKO 10 dpi vs. 20 dpi	*p* = 0.0125
					DKO 10 dpi vs. 30 dpi	*p* < 0.0001
					DKO 20 dpi vs. 30 dpi	*p* = 0.0036
r	2*M*, P23	Normal distribution	Two-way ANOVA; Tukey *post hoc* analysis	SN P23 ablation: NF186 intensity		
					Effect of genotype	*p* = 0.0007
					Effect of timing postinjection	*p* = 0.0308
					Genotype × timing interaction	*p* = 0.0308
					Control 10 dpi vs. DKO 10 dpi	*p* = 0.9972
					Control 20 dpi vs. DKO 20 dpi	*p* = 0.2550
					Control 60 dpi vs. DKO 60 dpi	*p* = 0.0029
					DKO 10 dpi vs. 20 dpi	*p* = 0.5495
					DKO 10 dpi vs. 60 dpi	*p* = 0.0070
					DKO 20 dpi vs. 60 dpi	*p* = 0.1800
s	2*M*, P93	Normal distribution	Two-way ANOVA; Tukey *post hoc* analysis	SN P93 ablation: NF186 intensity		
					Effect of genotype	*p* < 0.0001
					Effect of timing postinjection	*p* < 0.0001
					Genotype × timing interaction	*p* < 0.0001
					Control 10 dpi vs. DKO 10 dpi	*p* > 0.9999
					Control 20 dpi vs. DKO 20 dpi	*p* = 0.2330
					Control 120 dpi vs. DKO 120 dpi	*p* < 0.0001
					DKO 10 dpi vs. 20 dpi	*p* = 0.2696
					DKO 10 dpi vs. 120 dpi	*p* < 0.0001
					DKO 20 dpi vs. 120 dpi	*p* = 0.0003
t	2*M*′, P13	Normal distribution	Two-way ANOVA; Tukey *post hoc* analysis	SC P13 ablation: NF186 intensity		
					Effect of genotype	*p* < 0.0001
					Effect of timing postinjection	*p* = 0.0003
					Genotype × timing interaction	*p* = 0.0003
					Control 10 dpi vs. DKO 10 dpi	*p* = 0.9382
					Control 20 dpi vs. DKO 20 dpi	*p* = 0.0971
					Control 30 dpi vs. DKO 30 dpi	*p* < 0.0001
					DKO 10 dpi vs. 20 dpi	*p* = 0.5018
					DKO 10 dpi vs. 30 dpi	*p* < 0.0001
					DKO 20 dpi vs. 30 dpi	*p* = 0.0009
u	2*M*′, P23	Normal distribution	Two-way ANOVA; Tukey *post hoc* analysis	SC P23 ablation: NF186 intensity		
					Effect of genotype	*p* = 0.0013
					Effect of timing postinjection	*p* = 0.0112
					Genotype × timing interaction	*p* = 0.0113
					Control 10 dpi vs. DKO 10 dpi	*p* > 0.9999
					Control 20 dpi vs. DKO 20 dpi	*p* = 0.5115
					Control 60 dpi vs. DKO 60 dpi	*p* = 0.0010
					DKO 10 dpi vs. 20 dpi	*p* = 0.6657
					DKO 10 dpi vs. 60 dpi	*p* = 0.0028
					DKO 20 dpi vs. 60 dpi	*p* = 0.0329
v	2*M*′, P93	Normal distribution	Two-way ANOVA; Tukey *post hoc* analysis	SC P93 ablation: NF186 intensity		
					Effect of genotype	*p* = 0.0007
					Effect of timing postinjection	*p* < 0.0001
					Genotype × timing interaction	*p* < 0.0001
					Control 10 dpi vs. DKO 10 dpi	*p* > 0.9999
					Control 20 dpi vs. DKO 20 dpi	*p* = 0.9996
					Control 120 dpi vs. DKO 120 dpi	*p* < 0.0001
					DKO 10 dpi vs. 20 dpi	*p* = 0.9978
					DKO 10 dpi vs. 120 dpi	*p* < 0.0001
					DKO 20 dpi vs. 120 dpi	*p* < 0.0001
w	2*Q*	Normal distribution	Unpaired, two-tailed *t* test	SN P13 ablation: relative protein level		
					AnkG level: control vs. Ank cKO	*p* = 0.0002
					AnkG level: control vs. DKO	*p* = 0.006
					NF186 level: control vs. Ank cKO	*p* = 0.9415
					NF186 level: control vs. DKO	*p* = 0.0062
×	2*Q*′	Normal distribution	Unpaired, two-tailed *t* test	SC P13 ablation: relative protein level		
					AnkG level: control vs. Ank cKO	*p* = 0.0079
					AnkG level: control vs. DKO	*p* = 0.0013
					NF186 level: control vs. Ank cKO	*p* = 0.355
					NF186 level: control vs. DKO	*p* = 0.0003
y	2*R*	Normal distribution	Unpaired, two-tailed *t* test	SN P23 ablation: relative protein level		
					AnkG level: control vs. Ank cKO	*p* = 0.0404
					AnkG level: control vs. DKO	*p* = 0.0046
					NF186 level: control vs. Ank cKO	*p* = 0.3541
					NF186 level: control vs. DKO	*p* = 0.0249
z	2*R*′	Normal distribution	Unpaired, two-tailed *t* test	SC P23 ablation: relative protein level		
					AnkG level: control vs. Ank cKO	*p* = 0.0036
					AnkG level: control vs. DKO	*p* = 0.0016
					NF186 level: control vs. Ank cKO	*p* = 0.7327
					NF186 level: control vs. DKO	*p* = 0.0066
aa	2*S*	Normal distribution	Unpaired, two-tailed *t* test	SN P93 ablation: relative protein level		
					AnkG level: control vs. DKO	*p* = 0.0043
					NF186 level: control vs. DKO	*p* < 0.0001
ab	2*S*′	Normal distribution	Unpaired, two-tailed *t* test	SC P93 ablation: relative protein level		
					AnkG level: control vs. DKO	*p* = 0.0009
					NF186 level: control vs. DKO	*p* = 0.0002
ac	3*S*, P13	Normal distribution	Two-way ANOVA; Tukey *post hoc* analysis	SN P13 ablation: NaV intensity		
					Effect of genotype	*p* < 0.0001
					Effect of timing postinjection	*p* < 0.0001
					Genotype × timing interaction	*p* < 0.0001
					Control 10 dpi vs. DKO 10 dpi	*p* = 0.9992
					Control 20 dpi vs. DKO 20 dpi	*p* < 0.0001
					Control 30 dpi vs. DKO 30 dpi	*p* < 0.0001
					DKO 10 dpi vs. 20 dpi	*p* < 0.0001
					DKO 10 dpi vs. 30 dpi	*p* < 0.0001
					DKO 20 dpi vs. 30 dpi	*p* < 0.0001
ad	3*S*, P23	Normal distribution	Two-way ANOVA; Tukey *post hoc* analysis	SN P23 ablation: NaV intensity		
					Effect of genotype	*p* < 0.0001
					Effect of timing postinjection	*p* = 0.0002
					Genotype × timing interaction	*p* = 0.0002
					Control 10 dpi vs. DKO 10 dpi	*p* = 0.9996
					Control 20 dpi vs. DKO 20 dpi	*p* = 0.0003
					Control 60 dpi vs. DKO 60 dpi	*p* < 0.0001
					DKO 10 dpi vs. 20 dpi	*p* = 0.0011
					DKO 10 dpi vs. 60 dpi	*p* < 0.0001
					DKO 20 dpi vs. 60 dpi	*p* = 0.93009
ae	3*S*, P93	Normal distribution	Two-way ANOVA; Tukey *post hoc* analysis	SN P93 ablation: NaV intensity		
					Effect of genotype	*p* < 0.0001
					Effect of timing postinjection	*p* = 0.0027
					Genotype × timing interaction	*p* = 0.0027
					Control 10 dpi vs. DKO 10 dpi	*p* = 0.9996
					Control 20 dpi vs. DKO 20 dpi	*p* = 0.0058
					Control 120 dpi vs. DKO 120 dpi	*p* = 0.0012
					DKO 10 dpi vs. 20 dpi	*p* = 0.0032
					DKO 10 dpi vs. 120 dpi	*p* = 0.0004
					DKO 20 dpi vs. 120 dpi	*p* = 0.6551
af	3*T*, P13	Normal distribution	Two-way ANOVA; Tukey *post hoc* analysis	SC P13 ablation: NaV intensity		
					Effect of genotype	*p* < 0.0001
					Effect of timing postinjection	*p* = 0.3498
					Genotype × timing interaction	*p* = 0.3510
					Control 10 dpi vs. DKO 10 dpi	*p* = 0.0085
					Control 20 dpi vs. DKO 20 dpi	*p* = 0.0018
					Control 30 dpi vs. DKO 30 dpi	*p* = 0.0003
					DKO 10 dpi vs. 20 dpi	*p* = 0.9713
					DKO 10 dpi vs. 30 dpi	*p* = 0.2965
					DKO 20 dpi vs. 30 dpi	*p* = 0.7138
ag	3*T*, P23	Normal distribution	Two-way ANOVA; Tukey *post hoc* analysis	SC P23 ablation: NaV intensity		
					Effect of genotype	*p* < 0.0001
					Effect of timing postinjection	*p* = 0.0451
					Genotype × timing interaction	*p* = 0.0452
					Control 10 dpi vs. DKO 10 dpi	*p* = 0.4863
					Control 20 dpi vs. DKO 20 dpi	*p* = 0.0089
					Control 60 dpi vs. DKO 60 dpi	*p* = 0.0004
					DKO 10 dpi vs. 20 dpi	*p* = 0.2164
					DKO 10 dpi vs. 60 dpi	*p* = 0.0152
					DKO 20 dpi vs. 60 dpi	*p* = 0.8617
ah	3*T*, P93	Normal distribution	Two-way ANOVA; Tukey *post hoc* analysis	SC P93 ablation: NaV intensity		
					Effect of genotype	*p* = 0.0015
					Effect of timing postinjection	*p* = 0.0108
					Genotype × timing interaction	*p* = 0.0107
					Control 10 dpi vs. DKO 10 dpi	*p* = 0.9987
					Control 20 dpi vs. DKO 20 dpi	*p* = 0.7937
					Control 120 dpi vs. DKO 120 dpi	*p* = 0.0025
					DKO 10 dpi vs. 20 dpi	*p* = 0.9066
					DKO 10 dpi vs. 120 dpi	*p* = 0.0026
					DKO 20 dpi vs. 120 dpi	*p* = 0.0259
ai	3*U*	Normal distribution	Unpaired, two-tailed *t* test	SC- NaV: relative protein level		
					Control vs. P13 ablation DKO	*p* = 0.8532
					Control vs. P23 ablation DKO	*p* = 0.927
					Control vs. P93 ablation	*p* = 0.98
aj	4*S*, P13	Normal distribution	Two-way ANOVA; Tukey *post hoc* analysis	SN P13 ablation: BIV intensity		
					Effect of genotype	*p* < 0.0001
					Effect of timing postinjection	*p* = 0.0169
					Genotype × timing interaction	*p* = 0.0170
					Control 10 dpi vs. DKO 10 dpi	*p* = 0.1170
					Control 20 dpi vs. DKO 20 dpi	*p* = 0.0017
					Control 30 dpi vs. DKO 30 dpi	*p* < 0.0001
					DKO 10 dpi vs. 20 dpi	*p* = 0.1209
					DKO 10 dpi vs. 30 dpi	*p* = 0.0036
					DKO 20 dpi vs. 30 dpi	*p* = 0.6801
ak	4*S*, P23	Normal distribution	Two-way ANOVA; Tukey *post hoc* analysis	SN P23 ablation: BIV intensity		
					Effect of genotype	*p* < 0.0001
					Effect of timing postinjection	*p* = 0.0423
					Genotype × timing interaction	*p* = 0.0425
					Control 10 dpi vs. DKO 10 dpi	*p* = 0.7035
					Control 20 dpi vs. DKO 20 dpi	*p* = 0.1771
					Control 60 dpi vs. DKO 60 dpi	*p* = 0.0006
					DKO 10 dpi vs. 20 dpi	*p* = 0.9089
					DKO 10 dpi vs. 60 dpi	*p* = 0.0215
					DKO 20 dpi vs. 60 dpi	*p* = 0.1587
al	4*S*, P93	Normal distribution	Two-way ANOVA; Tukey *post hoc* analysis	SN P93 ablation: BIV intensity		
					Effect of genotype	*p* = 0.0006
					Effect of timing postinjection	*p* = 0.0394
					Genotype × timing interaction	*p* = 0.0394
					Control 10 dpi vs. DKO 10 dpi	*p* = 0.9642
					Control 20 dpi vs. DKO 20 dpi	*p* = 0.2846
					Control 120 dpi vs. DKO 120 dpi	*p* = 0.0040
					DKO 10 dpi vs. 20 dpi	*p* = 0.6927
					DKO 10 dpi vs. 120 dpi	*p* = 0.0147
					DKO 20 dpi vs. 120 dpi	*p* = 0.1642
am	4*T*, P13	Normal distribution	Two-way ANOVA; Tukey *post hoc* analysis	SC P13 ablation: BIV intensity		
					Effect of genotype	*p* < 0.0001
					Effect of timing postinjection	*p* = 0.2569
					Genotype × timing interaction	*p* = 0.2569
					Control 10 dpi vs. DKO 10 dpi	*p* = 0.0004
					Control 20 dpi vs. DKO 20 dpi	*p* < 0.0001
					Control 30 dpi vs. DKO 30 dpi	*p* < 0.0001
					DKO 10 dpi vs. 20 dpi	*p* = 0.9939
					DKO 10 dpi vs. 30 dpi	*p* = 0.2390
					DKO 20 dpi vs. 30 dpi	*p* = 0.5023
an	4*T*, P23	Normal distribution	Two-way ANOVA; Tukey *post hoc* analysis	SC P23 ablation: BIV intensity		
					Effect of genotype	*p* = 0.0003
					Effect of timing postinjection	*p* = 0.1459
					Genotype × timing interaction	*p* = 0.1459
					Control 10 dpi vs. DKO 10 dpi	*p* = 0.8248
					Control 20 dpi vs. DKO 20 dpi	*p* = 0.1930
					Control 60 dpi vs. DKO 60 dpi	*p* = 0.0043
					DKO 10 dpi vs. 20 dpi	*p* = 0.8728
					DKO 10 dpi vs. 60 dpi	*p* = 0.0925
					DKO 20 dpi vs. 60 dpi	*p* = 0.5341
ao	4*T*, P93	Normal distribution	Two-way ANOVA; Tukey *post hoc* analysis	SC P93 ablation: BIV intensity		
					Effect of genotype	*p* = 0.0198
					Effect of timing postinjection	*p* = 0.0050
					Genotype × timing interaction	*p* = 0.0050
					Control 10 dpi vs. DKO 10 dpi	*p* > 0.9999
					Control 20 dpi vs. DKO 20 dpi	*p* = 0.9978
					Control 120 dpi vs. DKO 120 dpi	*p* = 0.0042
					DKO 10 dpi vs. 20 dpi	*p* = 0.9877
					DKO 10 dpi vs. 120 dpi	*p* = 0.0031
					DKO 20 dpi vs. 120 dpi	*p* = 0.0019
ap	4*U*	Normal distribution	Unpaired, two-tailed *t* test	SC- BIV: relative protein level		
					Control vs. P13 ablation DKO	*p* = 0.7971
					Control vs. P23 ablation DKO	*p* = 0.9601
					Control vs. P93 ablation	*p* = 0.8546
aq	5*S*, P13	Normal distribution	Two-way ANOVA; Tukey *post hoc* analysis	SN P13 ablation: AnkR intensity		
					Effect of genotype	*p* < 0.0001
					Effect of timing postinjection	*p* = 0.0001
					Genotype × timing interaction	*p* = 0.0001
					Control 10 dpi vs. DKO 10 dpi	*p* = 0.0005
					Control 20 dpi vs. DKO 20 dpi	*p* < 0.0001
					Control 30 dpi vs. DKO 30 dpi	*p* > 0.9999
					DKO 10 dpi vs. 20 dpi	*p* = 0.0926
					DKO 10 dpi vs. 30 dpi	*p* = 0.0009
					DKO 20 dpi vs. 30 dpi	*p* < 0.0001
ar	5*S*, P23	Normal distribution	Two-way ANOVA; Tukey *post hoc* analysis	SN P23 ablation: AnkR intensity		
					Effect of genotype	*p* < 0.0001
					Effect of timing postinjection	*p* < 0.0001
					Genotype × timing interaction	*p* < 0.0001
					Control 10 dpi vs. DKO 10 dpi	*p* < 0.0001
					Control 20 dpi vs. DKO 20 dpi	*p* < 0.0001
					Control 60 dpi vs. DKO 60 dpi	*p* = 0.8245
					DKO 10 dpi vs. 20 dpi	*p* < 0.0001
					DKO 10 dpi vs. 60 dpi	*p* < 0.0001
					DKO 20 dpi vs. 60 dpi	*p* < 0.0001
as	5*S*, P93	Normal distribution	Two-way ANOVA; Tukey *post hoc* analysis	SN P93 ablation: AnkR intensity		
					Effect of genotype	*p* < 0.0001
					Effect of timing postinjection	*p* < 0.0001
					Genotype × timing interaction	*p* < 0.0001
					Control 10 dpi vs. DKO 10 dpi	*p* = 0.0008
					Control 20 dpi vs. DKO 20 dpi	*p* < 0.0001
					Control 120 dpi vs. DKO 120 dpi	*p* = 0.1776
					DKO 10 dpi vs. 20 dpi	*p* < 0.0001
					DKO 10 dpi vs. 120 dpi	*p* < 0.0001
					DKO 20 dpi vs. 120 dpi	*p* < 0.0001
at	5*T*, P13	Normal distribution	Two-way ANOVA; Tukey *post hoc* analysis	SC P13 ablation: AnkR intensity		
					Effect of genotype	*p* < 0.0001
					Effect of timing postinjection	*p* < 0.0002
					Genotype × timing interaction	*p* < 0.0003
					Control 10 dpi vs. DKO 10 dpi	*p* = 0.3295
					Control 20 dpi vs. DKO 20 dpi	*p* < 0.0001
					Control 30 dpi vs. DKO 30 dpi	*p* = 0.9979
					DKO 10 dpi vs. 20 dpi	*p* < 0.0001
					DKO 10 dpi vs. 30 dpi	*p* = 0.4570
					DKO 20 dpi vs. 30 dpi	*p* < 0.0001
au	5*T*, P23	Normal distribution	Two-way ANOVA; Tukey *post hoc* analysis	SC P23 ablation: AnkR intensity		
					Effect of genotype	*p* < 0.0001
					Effect of timing postinjection	*p* < 0.0001
					Genotype × timing interaction	*p* < 0.0001
					Control 10 dpi vs. DKO 10 dpi	*p* = 0.8577
					Control 20 dpi vs. DKO 20 dpi	*p* < 0.0001
					Control 60 dpi vs. DKO 60 dpi	*p* > 0.9999
					DKO 10 dpi vs. 20 dpi	*p* < 0.0001
					DKO 10 dpi vs. 60 dpi	*p* = 0.8179
					DKO 20 dpi vs. 60 dpi	*p* < 0.0001
av	5*T*, P93	Normal distribution	Two-way ANOVA; Tukey *post hoc* analysis	SC P93 ablation: AnkR intensity		
					Effect of genotype	*p* < 0.0001
					Effect of timing postinjection	*p* < 0.0001
					Genotype × timing interaction	*p* < 0.0001
					Control 10 dpi vs. DKO 10 dpi	*p* = 0.8502
					Control 20 dpi vs. DKO 20 dpi	*p* < 0.0001
					Control 120 dpi vs. DKO 120 dpi	*p* = 0.4611
					DKO 10 dpi vs. 20 dpi	*p* < 0.0001
					DKO 10 dpi vs. 120 dpi	*p* = 0.9796
					DKO 20 dpi vs. 120 dpi	*p* < 0.0001
aw	5*U*	Normal distribution	Unpaired, two-tailed *t* test	SC- AnkR: relative protein level		
					Control vs. P13 ablation DKO	*p* = 0.9677
					Control vs. P23 ablation DKO	*p* = 0.4293
					Control vs. P93 ablation	*p* = 0.2763
ax	6*B*	Normal distribution	Two-way ANOVA; Tukey *post hoc* analysis	P13 ablation: NCV		
					Effect of genotype	*p* = 0.0927
					Effect of timing postinjection	*p* < 0.0001
					Genotype × timing interaction	*p* < 0.0001
					Control 10 dpi vs. DKO 10 dpi	*p* = 0.0005
					Control 20 dpi vs. DKO 20 dpi	*p* < 0.0001
					Control 30 dpi vs. DKO 30 dpi	*p* < 0.0001
					DKO 10 dpi vs. 20 dpi	*p* = 0.7586
					DKO 10 dpi vs. 30 dpi	*p* = 0.2018
					DKO 20 dpi vs. 30 dpi	*p* = 0.9264
ay	6*C*	Normal distribution	Two-way ANOVA; Tukey *post hoc* analysis	P13 ablation: Notch Amp		
					Effect of genotype	*p* = 0.0006
					Effect of timing postinjection	*p* < 0.0001
					Genotype × timing interaction	*p* = 0.0016
					Control 10 dpi vs. DKO 10 dpi	*p* = 0.9992
					Control 20 dpi vs. DKO 20 dpi	*p* = 0.3675
					Control 30 dpi vs. DKO 30 dpi	*p* < 0.0001
					DKO 10 dpi vs. 20 dpi	*p* = 0.7221
					DKO 10 dpi vs. 30 dpi	*p* = 0.9993
					DKO 20 dpi vs. 30 dpi	*p* = 0.4799
az	6*E*	Normal distribution	Two-way ANOVA; Tukey *post hoc* analysis	P23 ablation: NCV		
					Effect of genotype	*p* < 0.0001
					Effect of timing postinjection	*p* = 0.0428
					Genotype × timing interaction	*p* = 0.0171
					Control 10 dpi vs. DKO 10 dpi	*p* > 0.9999
					Control 20 dpi vs. DKO 20 dpi	0.0021
					Control 60 dpi vs. DKO 60 dpi	0.0004
					DKO 10 dpi vs. 20 dpi	*p* > 0.9999
					DKO 10 dpi vs. 60 dpi	*p* = 0.9991
					DKO 20 dpi vs. 60 dpi	*p* = 0.9998
ba	6*F*	Normal distribution	Two-way ANOVA; Tukey *post hoc* analysis	P23 ablation: Notch Amp		
					Effect of genotype	*p* = 0.0007
					Effect of timing postinjection	*p* = 0.5107
					Genotype × timing interaction	*p* = 0.4310
					Control 10 dpi vs. DKO 10 dpi	*p* = 0.9582
					Control 20 dpi vs. DKO 20 dpi	*p* = 0.0492
					Control 60 dpi vs. DKO 60 dpi	*p* = 0.034
					DKO 10 dpi vs. 20 dpi	*p* = 0.6092
					DKO 10 dpi vs. 60 dpi	*p* = 0.8662
					DKO 20 dpi vs. 60 dpi	*p* = 0.9906
bb	6*H*	Normal distribution	Two-way ANOVA; Tukey *post hoc* analysis	P93 ablation: NCV		
					Effect of genotype	*p* < 0.0001
					Effect of timing postinjection	*p* = 0.9269
					Genotype × timing interaction	*p* = 0.0770
					Control 20 dpi vs. DKO 20 dpi	*p* = 0.8318
					Control 60 dpi vs. DKO 60 dpi	*p* = 0.0002
					Control 120 dpi vs. DKO 120 dpi	*p* = 0.0644
					DKO 20 dpi vs. 60 dpi	*p* = 0.9163
					DKO 20 dpi vs. 120 dpi	*p* = 0.4341
					DKO 60 dpi vs. 120 dpi	*p* = 0.9652
bc	6*I*	Normal distribution	Two-way ANOVA; Tukey *post hoc* analysis	P93 ablation: Notch Amp		
					Effect of genotype	*p* = 0.0213
					Effect of timing postinjection	*p* = 0.0088
					Genotype × timing interaction	*p* = 0.0174
					Control 20 dpi vs. DKO 20 dpi	*p* > 0.9999
					Control 60 dpi vs. DKO 60 dpi	*p* = 0.0713
					Control 120 dpi vs. DKO 120 dpi	*p* = 0.0041
					DKO 20 dpi vs. 60 dpi	*p* > 0.9999
					DKO 20 dpi vs. 120 dpi	*p* = 0.3463
					DKO 60 dpi vs. 120 dpi	*p* = 0.4148

Note: GraphPad Prism, which was used to perform all statistical analyses, does not report exact *p*-values higher than 0.9999 or lower than 0.0001.

All statistical tests are summarized with exact *p*-values.

### *In vivo* recordings

At various time points post-tamoxifen, *in vivo* nerve conduction velocities (NCVs) and amplitudes were recorded from the sciatic nerve of aged matched control and double AnkG/NF186 cKO littermates. The mice were anesthetized by continuous isoflurane (5% aerosolized), and electrophysiological recordings were collected using a Nicolet Teca Synergy portable neurologic system (Natus Neurology) as previously described (Taylor et al., 2017). Briefly, the recording electrodes were placed in the dorsum of the foot, and two separate recordings were made: at the ankle (0.02 ms, 4 mA) and the sciatic notch (0.02 ms, 8 mA). NCV studies were limited to the left sciatic nerve so that the right sciatic nerve could be used for further analyses.

### Immunofluorescence

At various time points post-tamoxifen, control, single AnkG cKO, single NF186 cKO, and double AnkG/NF186 cKO littermates were anesthetized through an i.p. injection of Avertin (2% 2-2-2 tribromoethanol in 2-methyl-2-butanol). The right sciatic nerve was removed and fixed for 30 min in 4% paraformaldehyde (PFA) in 0.01 m PBS. Then the mouse was perfused intracardially using a peristaltic pump for 3 min with saline followed by 2 min with a chilled 1% PFA, 1% sucrose solution in 0.1 m phosphate buffer (PB). Spinal cords were harvested, postfixed for 2 h at 4°C in the 1% PFA solution, and then allowed to submerge in 30% sucrose before being cryosectioned into 14-µm sections. After PBS washes, the sciatic nerve was teased on the slides and allowed to dry overnight. Immunostaining of both spinal cord slices and teased sciatic nerves was performed as previously described (Taylor et al., 2017).

### Image analysis

Confocal images were acquired with a Zeiss LSM 710 Microscope using a 40× oil objective as previously described (Taylor et al., 2017). Briefly, identical settings were used to capture images from control and mutant samples, and images shown are maximal-intensity projections from Z-stacks with 0.4-µm interval. For quantification of nodal intensities, three z-stack images were taken for each mouse, and a minimum of 50 nodes for each nodal marker per tissue were quantified. All values were arithmetically adjusted to yield a unit of 1 for the control group at each time point.

### Immunoblotting

Sciatic nerve and spinal cord were collected from anesthetized control, single AnkG cKO, and double AnkG/NF186 cKO littermates at various time points post-tamoxifen and stored at –80˚C until processing. Tissues were homogenized in ice-cold RIPA buffer (25 mm Tris-HCl, pH 7.5, 150 mm NaCl, 1 mm EDTA, 1% NP-40, and 5% glycerol) with protease inhibitors (#A32953, Thermo Fisher Scientific) and phosphatase inhibitor mix (RRID:AB_10189608, #sc-45044, Santa Cruz Biotechnology). The lysates were sonicated for 10 s before centrifugation at 13,000 × *g* at 4°C for 30 min. The supernatant was then mixed with 6× sample buffer and heated to 37°C, and immunoblotting was conducted as previously described (Saifetiarova et al., 2017). When using IR-conjugated secondary antibodies (1:10000 for 1 h), membranes were imaged using an Odyssey scanner (LI-COR).

The intensities of Western blot bands were quantified using ImageJ software (RRID:SCR_003070; NIH) and normalized to β-actin or GAPDH loading controls. Specifically, for AnkG immunoblots, the 270- and 480-kDa band intensities from each animal were added together and are reported as a single relative protein level. The normalized intensity of each protein within each group was calculated by averaging band intensity from three mice, and representative blots for each protein are shown.

### Electron microscopy

Double AnkG/NF186 cKO and controls at the specified time points post-tamoxifen were anesthetized and perfused intracardially using a peristaltic pump with saline for 10 min followed by a 5% glutaraldehyde:4% PFA solution prepared in sodium cacodylate buffer for 30 min. After perfusion, the whole animal was submerged in the 5%/4% fixative for at least 1 week before the spinal cord and sciatic nerve were dissected. The tissues were cut into 1-mm square pieces and processed as described (Green et al., 2013; Taylor et al., 2017). Once embedded in Polybed, the blocks were submitted to the UTHSCSA Electron Microscopy Lab for sectioning and contrast-staining. Grids were imaged on a JEOL 1230 transmission electron microscope using an Advanced Microscopy Techniques camera and software (Woburn, MA).

## Results

### Simultaneous loss of neurofascin 186 with ankyrin G dramatically reduces lifespan

**Figure 1.**
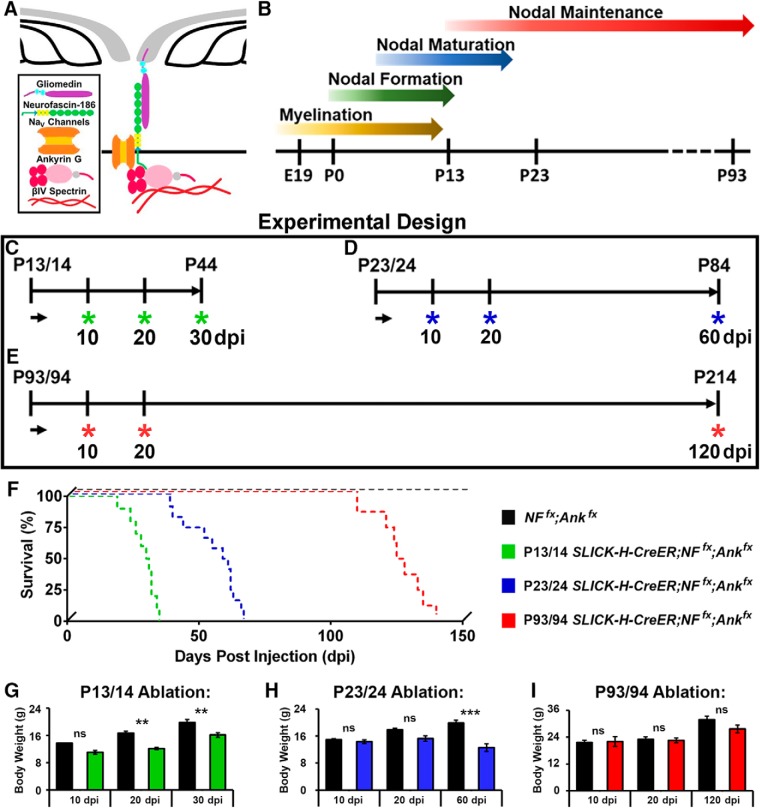
Timing of NF186/AnkG ablation alters survival. ***A***, Schematic diagram of key proteins involved in the organization and/or stabilization of nodes of Ranvier within myelinated fibers in the PNS. ***B***, Timeline of myelination, nodal formation, maturation, and nodal maintenance in mice. ***C–E***, Schematic representation of tamoxifen injections schemes for *Ank3* and *Nfasc^NF186^* ablation from myelinated axons. *SLICK-H-CreER;NF^fx^;Ank^fx^* and *NF^fx^;Ank^fx^* control mice were injected at P13/14 (***C***), P23/24 (***D***), or P93/94 (***E***) and analyzed 10, 20, 30, 60, or 120 dpi. (F) Survival curve representing the numbers of days lived after tamoxifen ablation for *SLICK-H-CreER;NF^fx^;Ank^fx^* mice injected at P13/14 (green line), P23/24 (blue line), or P93/94 (red line) compared to age-matched *NF^fx^;Ank^fx^* littermates (black line). Each curve is significantly different (*p* < 0.0001) from each other by Mantel–Cox log rank test, Bonferroni *post hoc* analysis (*n* = 8–10 mice/group). ***G–I***, Graph representing the body weight of *NF^fx^;Ank^fx^* (black bar) and *SLICK-H-CreER;NF^fx^;Ank^fx^* mice injected at P13/14 (***G***, green bar), P23/24 (***H***, blue bar), or P93/94 (***I***, red bar). Black asterisks indicate statistical differences between control and mutant at each time point by two-way ANOVA, Tukey *post hoc* analysis (*n* = 8–10 mice/group). Data are represented as mean ± SEM.

A unique molecular interplay between cell adhesion and cytoskeletal scaffolding proteins drives the clustering of ion channels at nodes of Ranvier along myelinated axons. While the cytoskeletal component, AnkG, binds to βIV Spectrin, Na_V_ channels, and NF186 intracellularly, the transmembrane NF186 extends beyond the cell membrane, interacting with glial extracellular matrix proteins (depicted in Fig. 1*A*). Although the individual roles of these proteins in the initial formation of the node have been well studied, their roles in nodal stability have only recently begun to be elucidated (Saifetiarova et al., 2017; Taylor et al., 2017). The combined contributions of AnkG and NF186 in nodal maintenance and what impact their combined loss would have on the nodal domain as well as the animal lifespan has not been addresesd.

To study the combined contributions of AnkG [encoded by *Ankyrin 3 (Ank3)*] and neuronal Nfasc^NF186^ to the stability of the nodal complex at different developmental stages that coincide with myelination and axonal domain organization (Fig. 1*B*), we crossed *Ank3^Flox^* (*Ank^fx^*) mice (Paez-Gonzalez et al., 2011) with *Nfasc^Flox^* (*NF^fx^*) mice (Pillai et al., 2009) to generate double *NF^fx^;Ank^fx^*. This double floxed line was crossed with *SLICK-H* mice, which use a *Thy1.2* promoter modified to be specifically expressed in neurons (Caroni, 1997; Young et al., 2008). Thus, genotypes of control (*NF^fx^;Ank^fx^*), single AnkG cKO (*SLICK-H;Ank^fx^*), single NF186 cKO (*SLICK-H;NF^fx^*), and double AnkG/NF186 cKO (*SLICK-H;NF^fx^;Ank^fx^*) were generated.

Controls, single, and double AnkG/NF186 cKO littermates were given tamoxifen injections for 2 consecutive days starting at P13 to specifically ablate AnkG and/or NF186 from neurons after the initial clustering of nodes of Ranvier had begun during the stage of nodal maturation (Fig. 1*C*). These mice were examined at 10, 20, and 30 days post injection (dpi), as P13/14 ablated *SLICK-H;NF^fx^;Ank^fx^* rarely survived past 35 dpi (Fig. 1*F*). As early as 5 dpi, double AnkG/NF186 cKO were distinguishable from their littermates by altered gait and tremor. By 20 dpi, these phenotypes progressed to include significantly reduced body weight in the double AnkG/NF186 cKO at 12.2 ± 0.4 g compared to matched controls at 16.7 ± 0.5 g (Fig. 1*G*). By 30 dpi, surviving double AnkG/NF186 cKO mice displayed hindlimb clasping, kyphosis, and paresis, which was never observed in tamoxifen-injected controls and did not begin to appear in P13/14 single-ablated AnkG or NF186 cKO until after 30 dpi.

To further examine the combined roles of AnkG and NF186 in the maintenance of nodes after nodal clustering is complete, controls, single, and double AnkG/NF186 cKOs littermates were injected with tamoxifen for 2 consecutive days starting at P23 during adolescence (Fig. 1*D*). These mice were examined at 10, 20, and 60 dpi, as *SLICK-H;NF^fx^;Ank^fx^* injected at P23/24 did not survive beyond an average of 60 dpi (Fig. 1*F*). This median survival age was significantly longer than those ablated at P13/14, which showed a median survival age of 30.5 dpi (χ^2^ = 25.28; *p* < 0.0001, Mantel–Cox). By 10 dpi, double AnkG/NF186 cKOs ablated at P23/24 displayed a defined tremor, which developed into an altered gait and hindlimb clasping by 20 dpi. Although the phenotype was delayed compared to the P13/14 injected double cKOs, those double cKOs injected at P23 ultimately showed partial hindlimb paralysis and severe spinal deformation by 60 dpi, which as previously reported was not seen until 6 months postinjection (mpi) in *SLICK-H;NF^fx/-^* and 8mpi in *SLICK-H;Ank^fx/-^* (Saifetiarova et al., 2017; Taylor et al., 2017). In addition to ataxic phenotype and shortened lifespan, a significant reduction in body weight was observed at 60 dpi in P23/24 ablated double cKOs (12.6 ± 1.1 g) compared to matched controls (19.9 ± 0.8 g; Fig. 1*H*). Together these initial results reveal that despite the timing of ablation, combined loss of the cell adhesion molecule NF186 with the cytoskeletal component AnkG leads to an enhanced phenotype and ultimately quicker demise of the animals compared to single ablation of NF186 or AnkG, which suggest a synergistic function of these proteins. Furthermore, delaying the stage at which ablation of AnkG and NF186 occurred by just 10 days led to essentially a doubling of the survival postinjection.

To thoroughly examine nodal stability at different stages, we next asked if delaying ablation of AnkG and NF186 to a time point later in adult life would lead to any further change in the kinetics of nodal destabilization, as one might hypothesize that once a node has fully matured, no changes would be seen in the timeline. Controls and double AnkG/NF186 cKO littermates were injected with tamoxifen for 2 consecutive days starting at P93/94 (Fig. 1*E*). These mice were examined at 10, 20, and 120 dpi, as *SLICK-H;NF^fx^;Ank^fx^* ablated at P93 had a median survival rate of 126.5 dpi, which was significantly longer than those injected at P13/14 and P23/24 (Fig. 1*F*; χ^2^ = 17.72 and 18.68, respectively; *p* < 0.0001, Mantel–Cox). The double AnkG/NF186 cKOs were indistinguishable compared to control littermates at 30 dpi and did not begin to display tremor or altered gait until 60 dpi. By endpoint, the P93 ablated double cKOs showed significant kyphosis, hindlimb clasping, and paresis. Although the double AnkG/NF186 cKOs ablated at P93/94 ultimately displayed ataxia, these mice did not show any significant reduction in body weight compared to littermate controls, unlike the double cKOs ablated at P13/14 and P23/24 (Fig. 1*I*). Together, these data indicate that loss of AnkG/NF186 at ∼3 months of adult life has a less dramatic impact on survival than at early stages and further suggest nodal disorganization may occur at different rates depending on the age of the node.

### Neurofascin 186 and AnkG show differential stability during nodal maturation and maintenance

**Figure 2.**
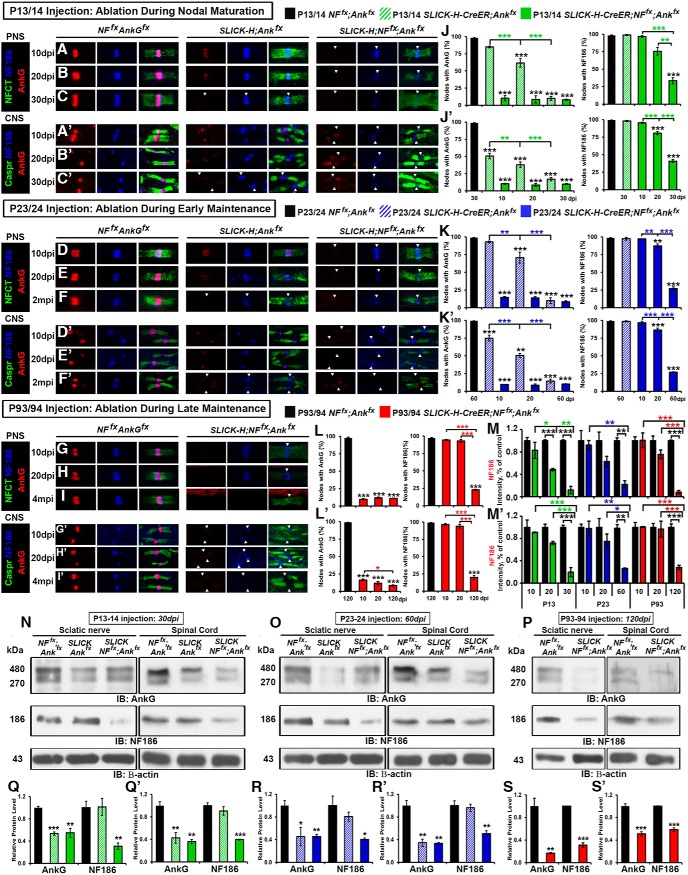
Combined ablation of NF186/AnkG reduces the nodal stability of AnkG and NF186. ***A–C′***, After tamoxifen ablation during nodal maturation (P13/14), SN fibers and SCs from *NF^fx^;Ank^fx^*, *SLICK-H-CreER;Ank^fx^,* and *SLICK-H-CreER;NF^fx^;Ank^fx^* were harvested at 10, 20, and 30 dpi. ***D–F′***, After tamoxifen ablation during early nodal maintenance (P23/24), SN fibers and SCs from *NF^fx^;Ank^fx^*, *SLICK-H-CreER;Ank^fx^*, and *SLICK-H-CreER;NF^fx^;Ank^fx^* were harvested at 10, 20, and 60 dpi. ***G–I′***, After tamoxifen ablation during late nodal maintenance (P23/24), SN fibers and SCs from *NF^fx^;Ank^fx^* and *SLICK-H-CreER;NF^fx^;Ank^fx^* were harvested at 10, 20, and 120 dpi. All SNs (***A–I***) were teased and immunostained with antibodies against AnkG (red), NF186 (blue), and NFCT (green); while SCs (***A′–I′***) were immunostained with antibodies against AnkG (red), NF186 (blue), and Caspr (green). Arrowheads mark AnkG-negative nodes. ***J–L′***, Quantification of the percentage of nodes with remaining AnkG and NF186 at various time points in *NF^fx^;Ank^fx^* (black bar), *SLICK-H-CreER;Ank^fx^* (striped green or blue bar), and *SLICK-H-CreER;NF^fx^;Ank^fx^* (solid green, blue, or red bar) SN (***J–L***) or SC (***J′–L′***). ***M***, ***M′***, Graphs representing intensity quantification of NF186 in the SN (***M***) and SC (***M′***) nodal area of *SLICK-H-CreER;NF^fx^;Ank^fx^* mice injected at P13/14 (green bars), P23/24 (blue bars), or P93/94 (red bars) normalized to age-matched *NF^fx^;Ank^fx^* control values (black bars). ***N–P***, Immunoblot analysis of SN and SC lysates from *NF^fx^;Ank^fx^* littermates and *SLICK-H-CreER;NF^fx^;Ank^fx^* mice injected at P13/14 (30 dpi), P23/24 (60 dpi), or P93/94 (120 dpi) with antibodies against AnkG, NF186, and β-actin. ***Q–S′***, Quantification of immunoblots relative to β-actin from the SN (***Q–S***) or SC (***Q′–S′***) lysates. All data are represented as mean ± SEM. Black asterisks indicate statistical differences between control and mutant; colored asterisks signify differences between time points among mutants. For immunostaining: *n* = 3–4 mice/group; 50–100 nodes per mouse; two-way ANOVA, Tukey *post hoc* analysis. For immunoblots: *n* = 3 mice; two-tailed Student’s *t* tests. Scale bar, 2 µm.

To confirm the ablation of AnkG and NF186 in the single and double AnkG/NF186 cKOs compared to controls, as well as to determine any difference in stability due to the timing of ablation, sciatic nerves (SNs) and spinal cords (SCs) at various time points were triple immunostained with antibodies against AnkG (red), NF186 (blue), and pan-Neurofascin (NFCT, which detects paranodal NF155 and nodal NF186) or Caspr, respectively (green, Fig. 2). As is seen in the *NF^fx^;Ank^fx^* column at all ages, control mice have a tight clustering of AnkG colocalized with NF186 at the node surrounded by the flanking paranodes in the PNS (Fig. 2*A–I*), which is sometimes flanked with a less intense paranodal AnkG staining in the CNS (Fig. 2*A′–I′* ), due to the oligodendrocyte expression of AnkG, as was previously reported (Chang et al., 2014).

After ablation at P13/14 during nodal maturation, single *SLICK-H;Ank^fx^* showed a significant increase in the number of nodes, with no detectable levels of AnkG in SN over time from 14.4 ± 2.6% at 10 dpi to 88.2 ± 2.9% at 30 dpi; however, there was no change in the number of nodes with NF186, which remained at control levels (Fig. 2*A–C*, *J*). In contrast, after P13 ablation, double *SLICK-H;NF^fx;^Ank^fx^* had no change in the number of nodes lacking AnkG, which was already 89.4 ± 3.8% at 10 dpi, and instead showed a significant increase in nodes with no detectable levels of NF186 from 1.8 ± 1.3% at 10 dpi to 65.0 ± 4.4% at 30 dpi (Fig. 2*J*). Although the number of nodes without detectable NF186 (65.0 ± 4.4%) had not dropped to the same percentage as nodes found without AnkG (90.4 ± 0.8%) by 30 dpi in the double AnkG/NF186 cKO, a significantly reduced intensity level of NF186 (12.0%) was measured at the nodes compared to age-matched controls (Fig. 2*M*). As in the PNS, P13/14 ablated single *SLICK-H;Ank^fx^* showed increasing amounts of AnkG-negative nodes in the SC from 48.7 ± 0.7% at 10 dpi to 82.4 ± 2.5% at 30 dpi, but no significant change in the number of nodes without NF186 (Fig. 2*A′–C′*, *J′* ). Although in the SC the number of nodes without AnkG in double cKOs was >90% by 10 dpi and remained steady, the nodes without detectable NF186 increased from 3.64 ± 0.9% at 10 dpi to 58.6 ± 2.1% at 30 dpi. The remaining intensity of NF186 at SC nodes 30 dpi after P13 ablation in the double AnkG/NF186 cKO was only 19.6% compared to control levels (Fig. 2*M′* ). Although single NF186 cKOs were included in these analyses as was previously reported, no significant loss of NF186 or AnkG was observed before 30 dpi in either PNS or CNS (Taylor et al., 2017; data not shown). Taken together, these results demonstrate that AnkG and NF186 have differential stability at nodes of Ranvier, with AnkG being fully depleted within just 10 days of ablation, while NF186 still remained at over 30% of nodes, albeit at very low levels at 30 dpi. Futhermore, these results indicate that combined ablation of AnkG with NF186 enhances their loss at nodes of Ranvier compared to individual ablation of AnkG or NF186.

Just as after P13/14 ablation, loss of AnkG alone at P23/24 during early maintenance resulted in a significant increase in the number of nodes without AnkG over time, leading to 89.3 ± 4.1% in the PNS and 85.2 ± 2.7% in the CNS at 60 dpi (Fig. 2*D–F*, *K*, *D′–F′*, *K′* ). Likewise, ablation of AnkG/NF186 in combination at P23/24 resulted in a majority of nodes without any detectable AnkG as early as 10 dpi in both tissues, but a gradual reduction of NF186 over time, leading to 71.8 ± 2.4% and 72.5 ± 0.5% of nodes with no detectable NF186 at 60 dpi in the PNS and CNS, respectively (Fig. 2*K*, *K′* ). Consistently, the intensity levels of NF186 gradually decreased to 22.2% in SN and 26.2% in SC at 60 dpi in the PNS and CNS, respectively, compared to controls (Fig. 2*M*, *M′* ). After P93/94 ablation during late maintenance, the level of nodes with undetectable AnkG was already significant in double AnkG/NF186 cKO by 10 dpi in both SN and SC; however, in SC, there were more AnkG-deficient nodes from 82.6 ± 1.3% at 10 dpi to 90.7 ± 0.8% at 30 dpi (Fig. 2*G–I*, *L*, *G′–I′, L′* ). In both SN and SC, the number of nodes without NF186 remained insignificant at control levels until 20 dpi and then gradually reached to 75.6 ± 0.9% and 79.4 ± 2.9% by 120 dpi, respectively (Fig. 2*L*, *L′* ).

As further verification of the ablation of AnkG and NF186, immunoblots were performed in SC and SN at the final time points: 30 dpi after P13/14 ablation, 60 dpi after P23/24 ablation, and 120 dpi after P93/94 ablation (Fig. 2*N–P*). When immunoblots were quantified, *SLICK-H;Ank^fx^* single cKO showed a significant reduction in the levels of AnkG compared to age-matched control levels in both SN (Fig. 2*Q*, *R*) and SC (Fig. 2*Q′*, *R′* ), but no significant change in the levels of NF186 compared to controls. In contrast, all double *SLICK-H;NF^fx;^Ank^fx^* display significant loss of both AnkG and NF186 regardless of the timing of injection in both the PNS (Fig. 2*Q–S*) and CNS (Fig. 2*Q′–S′* ). Although the trends in reduction of AnkG and NF186 levels between P13/14, P23/24, and P93/94 ablation were ultimately the same, overall, delaying the timeline of ablation led to a longer half-life of NF186 in the double cKOs, which was consistent with the delay of the ataxic phenotype seen in P93/94 compared to P23/24 and P13/14 ablated double cKOs.

### Timing of AnkG and NF186 ablation has differential effects on sodium channel destabilization in the CNS and PNS

**Figure 3.**
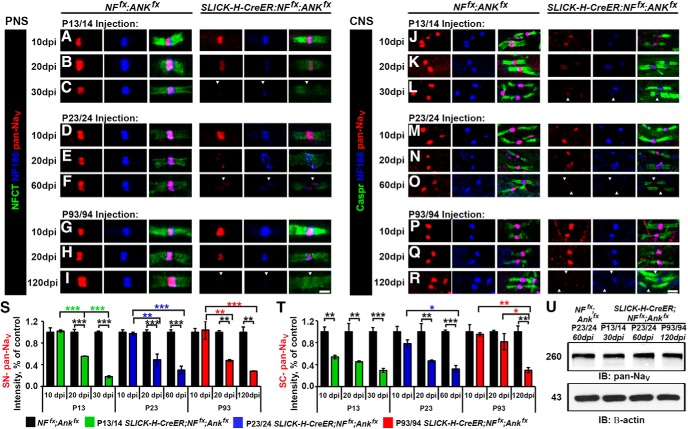
Timing of NF186/AnkG ablation alters stability of sodium channels in the CNS. ***A–I***, SN fibers from *NF^fx^;Ank^fx^* and *SLICK-H-CreER;NF^fx^;Ank^fx^* littermates injected at P13/14 (***A–C***, 10–30 dpi), P23/24 (***D–F***, 10–60 dpi), or P93/94 (***G–I***, 10–120 dpi) were triple immunostained with antibodies against pan-Na_V_ (red), NF186 (blue), and NFCT (green). ***J–R***, SCs from *NF^fx^;Ank^fx^* and *SLICK-H-CreER;NF^fx^;Ank^fx^* littermates injected at P13/14 (***J–L***, 10–30 dpi), P23/24 (***M–O***, 10–60 dpi), or P93/94 (***P–R***, 10–120 dpi) were triple immunostained with antibodies against pan-Na_V_ (red), NF186 (blue), and Caspr (green). ***S***, ***T***, Graphs representing intensity quantification of pan-Na_V_ in the SN (***S***) and SC (***T***) nodal area of *SLICK-H-CreER;NF^fx^;Ank^fx^* mice injected at P13/14 (green bars), P23/24 (blue bars), or P93/94 (red bars) normalized to age-matched *NF^fx^;Ank^fx^* control values (black bars). ***U***, Immunoblot analysis of SC lysates from 60 dpi *NF^fx^;Ank^fx^* injected at P23/24 compared to *SLICK-H-CreER;NF^fx^;Ank^fx^* mice injected at P13/14 (30 dpi), P23/24 (60 dpi), or P93/94 (120 dpi) with antibodies against pan-Na_V_ and β-actin. Arrowheads mark NF186-negative nodes. All data are represented as mean ± SEM (*n* = 3–4 mice/group; 50–100 nodes per mouse; two-way ANOVA, Tukey *post hoc* analysis). Black asterisks indicate statistical differences between control and mutant; colored asterisks signify differences between time points among mutants. Scale bar, 2 μm.

To determine the consequences of the combined loss of AnkG and NF186 on the maturation and maintenance of the nodal region, teased SNs from controls and double AnkG/NF186 cKOs at various time points postinjection were triple immunostained with antibodies against pan-Na_V_ (red), NF186 (blue), and NFCT (Fig. 3*A–I*). At 10 dpi, when NF186 was still present at the nodes without any appreciable loss in double AnkG/NF186 cKOs, there were no changes in either the localization or intensity of pan-Na_V_ channels in the PNS, no matter if ablation was performed at P13/14, P23/24, or P93/94 (Fig. 3*A*, *D*, *G*, *S*). By 20 dpi after P13/14 ablation of AnkG/NF186, when NF186 was undetectable in 26.3% of SN nodes and had dropped to an intensity below 50%, the level of Na_V_ channels was similarly reduced to 55.5% of the control intensity (Fig. 3*B*, *S*). Consistently, at 20 dpi after P23/24 and P93/94 ablation, the intensity of Na_V_ channels in double AnkG/NF186 cKOs was significantly reduced to 48.8% and 47.6% of the aged matched control, respectively (Fig. 3*E*, *H*, *S*). Scoring nodes with no detectable pan-Na_V_ at 30 dpi after P13/14 ablation, 60 dpi after P23/24, and 120 dpi after P93/94 revealed a 21.8 ± 4.4%, 40.6 ± 4.1%, and 28.7 ± 7.7% reduction in the double cKOs compared to matched controls, respectively; likewise, the intensity levels for pan-Na_V_ at nodes in double cKOs dropped below 30% of control levels at these terminal time points (Fig. 3*C*, *F*, *I*, *S*). Together, these data indicate that combined ablation of AnkG and NF186 leads to a rapid reduction of Na_V_ channels in the PNS, which is evident by 20 dpi despite the timing of ablation. This suggests that disruption of Na_V_ channels at least in the PNS is highly influenced by the loss of AnkG, which falls to below 10% by just 10 dpi and to a lesser extent NF186, which takes longer to dissipate from the node and varies depending on the timing of ablation.

To further study the effects of ablating AnkG and NF186 during nodal maturation versus maintenance in the CNS, SCs from double AnkG/NF186 cKOs and littermate controls were also immunostained with antibodies against pan-Na_V_ (red), NF186 (blue), and paranodal Caspr (Fig. 3*J–R*). At 10 dpi, when just as in SN, there was no appreciable loss of NF186 from the SC of double AnkG/NF186 cKOs, there was, however, a significant reduction in the intensity level of Na_V_ channels after P13/14 ablation at 53.8%, but not at P23/24 or P93/94 which remained at 78.1% and 95.3%, respectively (Fig. 3*J*, *M*, *P*, *T*). By 20 dpi, a significant reduction in intensity between controls and double cKO was seen after ablation of NF186 and AnkG at P13/14 and P23/24, but not at P93/94 (Fig. 3*J*, *M*, *P*, *T*). It is not until the final time point of 120 dpi after ablation of NF186 and AnkG at P93/94 that a significant reduction in the intensity of Na_V_ channels to below 30% is seen in the double cKO compared to controls (Fig. 3*R*, *T*). 30 dpi after P13/14 ablation and 60 dpi after P23/24 give similar reductions in Na_V_ channel intensity to ∼29.1% and 32% of control values in CNS (Fig. 3*L*, *O*, *T*). At these terminal time points in the CNS, there is also a significant decrease in the number of nodes with detectable pan-Na_V_ compared with controls to 58.2 ± 2.5% at 30 dpi after P13/14 ablation, 52.8.8 ± 4.7% 60 dpi after P23/24, and 34.3 ± 2.3% 120 dpi after P93/94. Despite the pronounced decrease of pan-Na_V_ levels at the nodes in *SLICK-H;NF^fx;^Ank^fx^* SC, there were no significant changes in the total protein levels of pan-Na_V_ channels normalized to β-actin compared to *NF^fx^;Ank^fx^* controls (Fig. 3*U*). Taken together, these data suggest that while the total level of Na_V_ channel is not decreased in the SC, Na_V_ channel does not maintain clustering at the node without the anchoring to cytoskeletal scaffold by AnkG, and the cell adhesion molecule, NF186. In addition, timing of ablation strongly influences the rate at which Na_V_ levels are lost from nodes in SC but not in SN, which may indicate that NF186 plays a greater role of anchoring Na_V_ levels in the CNS and is likely reflective of different extracellular binding partners between the PNS and CNS.

### βIV spectrin stability at the nodes increases with age

**Figure 4.**
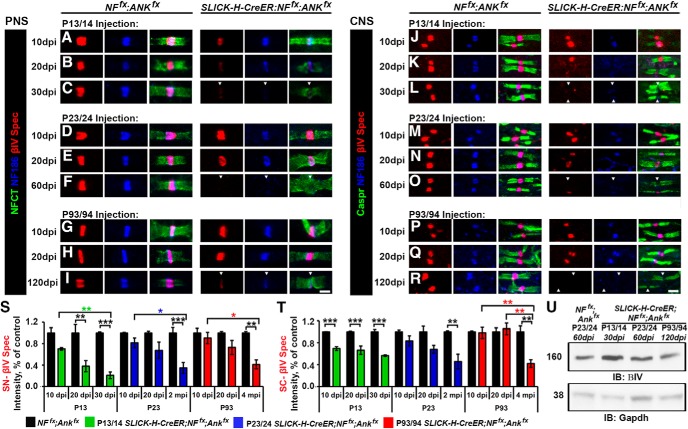
Nodal stability of βIV Spectrin is altered by the timing of NF186/AnkG ablation. ***A–I***, SN fibers from *NF^fx^;Ank^fx^,* and *SLICK-H-CreER;NF^fx^;Ank^fx^* littermates injected at P13/14 (***A–C***, 10–30 dpi), P23/24 (***D–F***, 10–60 dpi), or P93/94 (***G–I***, 10–120 dpi) were triple immunostained with antibodies against βIV Spectrin (red), NF186 (blue), and NFCT (green). ***J–R***, SCs from *NF^fx^;Ank^fx^* and *SLICK-H-CreER;NF^fx^;Ank^fx^* littermates injected at P13/14 (***J–L***, 10–30 dpi), P23/24 (***M–O***, 10–60 dpi), or P93/94 (***P–R***, 10–120 dpi) were triple immunostained with antibodies against βIV Spectrin (red), NF186 (blue), and Caspr (green). ***S***, ***T***, Graphs representing intensity quantification of βIV Spectrin in the SN (***S***) and SC (***T***) nodal area of *SLICK-H-CreER;NF^fx^;Ank^fx^* mice injected at P13/14 (green bars), P23/24 (blue bars), or P93/94 (red bars) normalized to age-matched *NF^fx^;Ank^fx^* control values (black bars). ***U***, Immunoblot analysis of SC lysates from 60 dpi *NF^fx^;Ank^fx^* injected at P23/24 compared to *SLICK-H-CreER;NF^fx^;Ank^fx^* mice injected at P13/14 (30 dpi), P23/24 (60 dpi), or P93/94 (120 dpi) with antibodies against βIV Spectrin and Gapdh. Arrowheads mark NF186-negative nodes. All data are represented as mean ± SEM (*n* = 3–4 mice/group; 50–100 nodes per mouse; two-way ANOVA, Tukey *post hoc* analysis). Black asterisks indicate statistical differences between control and mutant; colored asterisks signify differences between time points among mutants. Scale bar, 2 μm.

To thoroughly elucidate the impact of timing of ablation of AnkG and NF186 on nodal stabilization, teased SNs and SC slices from controls and double AnkG/NF186 cKOs at various time points postinjection were also triple immunostained with antibodies against βIV Spectrin (red), NF186 (blue), and NFCT or Caspr (green) in SN (Fig. 4*A–I*) and SC (Fig. 4*J–R*), respectively. At just 10 dpi after ablation at P13/14 when there is complete loss of AnkG but not yet loss of NF186 in double cKO, a trend toward a reduction in intensity of βIV Spectrin at SN nodes (70.2 ± 2.4%) and already a significant difference at SC nodes (69.5 ± 3.4%) is observed compared to controls (Fig. 4*A*, *J*, *S*, *T*). While in SN there is further reduction in βIV Spectrin intensity by 20 dpi to 38.3% and 30 dpi to 21.0%, in SC after P13/14 ablation there is no additional decrease in the intensity, which remains at 56.5% of control values at 30 dpi (Fig. 4*B*, *C*, *K*, *L*, *S*). Although there was not a significant loss of βIV Spectrin intensity over time after P13/14 ablation in SC, there was a significant decrease in the number of nodes with detectable levels of βIV Spectrin in both PNS and CNS to 63.9 ± 2.8 and 76.1 ± 4.5% by 30 dpi (Fig. 4*K*, *L*).

In contrast to ablation during nodal maturation, loss of NF186 with AnkG during either early or late nodal maintenance resulted in no significant changes in either the localization or intensity βIV Spectrin at 10 or 20 dpi in either tissue (Fig. 4*D–T*). Only at 60 dpi after P23/24 and 120 dpi after P93/94 ablation of NF186 and AnkG was a significant reduction detected in βIV intensity compared to controls (Fig. 4*F*, *I*, *O*, *R*–*T*). At these terminal time points, there was also a reduction in the number of nodes with detectable βIV Spectrin in both SN (62.7 ± 5.9 and 56.1 ± 4.0%) and SC (60.3 ± 7.9 and 49.6 ± 12.9%) for P23/24 and P93/94 ablation, respectively. Although the levels of nodal βIV Spectrin were reduced, there was no significant reduction in the total protein levels of βIV in *SLICK-H;NF^fx;^Ank^fx^* SC when normalized to GAPDH compared to *NF^fx^;Ank^fx^* controls at the terminal time point for P13/14, P23/24, and P93/94 ablation (Fig. 4*U*). Consistently, no reduction in total βIV Spectrin protein levels was seen in single AnkG or NF186 cKO which was previously reported for P23 injections (Saifetiarova et al., 2017; Taylor et al., 2017). Together these results show that the stability of βIV Spectrin in the nodal complex is dependent on interactions between both the cytoskeletal competent AnkG and the cell adhesion molecule NF186, as well as on the timing of ablation in either maturation or maintenance stages.

### AnkR is not sufficient to maintain nodal stability without AnkG and NF186

**Figure 5.**
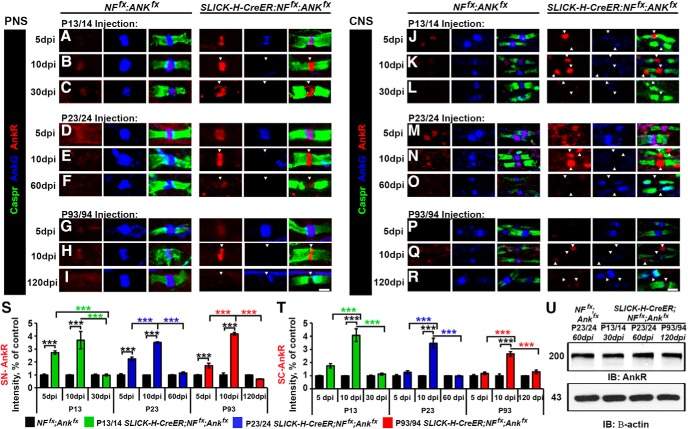
Combined ablation of NF186/AnkG reveals that AnkR fails to sustain nodal stability. ***A–I***, PNS fibers from *NF^fx^;Ank^fx^* and *SLICK-H-CreER;NF^fx^;Ank^fx^* littermates injected at P13/14 (***A–C***, 5–30 dpi), P23/24 (***D–F***, 5–60 dpi), or P93/94 (***G–I***, 5–120 dpi) were triple immunostained with antibodies against AnkR (red), NF186 (blue), and NFCT (green). ***J–R***, SCs from *NF^fx^;Ank^fx^* and *SLICK-H-CreER;NF^fx^;Ank^fx^* littermates injected at P13/14 (***J–L***, 5–30 dpi), P23/24 (***M–O***, 5-60 dpi), or P93/94 (***P–R***, 5–120 dpi) were triple immunostained with antibodies against AnkR (red), NF186 (blue), and Caspr (green). ***S***, ***T***, Graphs representing intensity quantification of AnkR in the PNS (***S***) and CNS (***T***) nodal area of *SLICK-H-CreER;NF^fx^;Ank^fx^* mice injected at P13/14 (green bars), P23/24 (blue bars), or P93/94 (red bars) normalized to age-matched *NF^fx^;Ank^fx^* control values (black bars). ***U***, Immunoblot analysis of SC lysates from 60 dpi *NF^fx^;Ank^fx^* injected at P23/24 compared to *SLICK-H-CreER;NF^fx^;Ank^fx^* mice injected at P13/14 (30 dpi), P23/24 (60 dpi), or P93/94 (120 dpi) with antibodies against AnkR and β-actin. Arrowheads mark Ank-G negative nodes. All data are represented as mean ± SEM (*n* = 3–4 mice/group; 50–100 nodes per mouse; two-way ANOVA, Tukey *post hoc* analysis). Black asterisks indicate statistical differences between control and mutant; while colored asterisks signify differences between time points among mutants. Scale bar, 2 μm.

Previously, loss of AnkG at the node was found to lead to a dramatic increase in Ankyrin R (AnkR), a resident nodal protein (Ho et al., 2014; Saifetiarova et al., 2017); however, when NF186 was ablated, a significant reduction in the level of AnkR was observed (Taylor et al., 2017). As our current model double AnkG/NF186 cKOs reflects loss of both proteins, we next determined what the consequences of ablating both AnkG and NF186 at different time windows would have on AnkR levels. PNS and CNS nerves from controls and double AnkG/NF186 cKOs at various time points postinjection were immunostained with antibodies against AnkR (red), AnkG (blue), and Caspr (green; Fig. 5). As AnkG is already depleted by 10 dpi after P13/14, P23/24, and P93/94 ablation of AnkG/NF186 in both PNS and CNS (Fig. 2), these studies were started at 5 dpi to capture a time point before AnkG has completely moved out of the node. Interestingly, at 5 dpi in both PNS and CNS, the levels of AnkG varied with timing of ablation, so that there was less remaining AnkG with P13/14 ablation compared to P23/24 and even less compared to P93/94 which appeared to be at control levels (Fig. 5*A–R*).

In the PNS at 5 dpi, AnkR levels quickly increased to compensate as AnkG was being depleted from the node, which is reflected by a 2.73-fold increase in AnkR levels after P13/14, 2.25-fold after P23/24, and 1.72-fold after P93/94 (Fig. 5*A*, *D*, *G*, *S*). By 10 dpi, as AnkG levels reached <10%, the intensity of AnkR continued to rise to >3.5-fold compared to control in all ablation schemes (Fig. 5*B*, *E*, *H*, *S*). At the terminal time points when not only AnkG but also NF186 is depleted from the node, AnkR levels fell back to or even below control levels (Fig. 5*C*, *F*, *I*, *S*). In addition, there was a reduction in the number of AnkR-positive nodes compared to controls to 86.1 ± 2.7% at P30 after P13/14 ablation, to 85.7 ± 2.5% at P60 after P23/24 ablation, and to 74.8 ± 0.9% at P120 after P93/94 ablation. In contrast to PNS, AnkR took longer to move into the nodes after AnkG was lost, which was reflected by no significant increase in AnkR intensity at 5 dpi, although there was a trend with a 1.75-fold increase compared to controls at 5 dpi after P13/14 ablation of NF186 and AnkG (Fig. 5*J*, *M*, *P*, *T*). At 10 dpi, AnkR intensity levels had significantly increased to 4.1-fold after P13/14, 3.5-fold after P23/24, and 2.7-fold after P93/94 compared to age-matched controls (Fig. 5*K*, *N*, *Q*, *T*). Just as in PNS, AnkR levels fell back to around control values by the final time point in SC whether ablation of AnkG/NF186 had occurred at P13/14, P23/24, or P93/94 (Fig. 5*L*, *O*, *R*, *T*). There were also very similar reductions in the number of AnkR-positive nodes compared to controls between PNS and CNS, with 85.6 ± 1.3% at P30 after P13/14 ablation, 83.6 ± 2.1% at P60 after P23/24 ablation, and 71.8 ± 4.3% at P120 after P93/94 ablation. Not surprisingly, due to the minimal loss of AnkR compared to controls at the final time point, there was neither a significant increase nor decrease in total protein levels of AnkR in *SLICK-H;NF^fx;^Ank^fx^* SC normalized to β-actin compared to *NF^fx^;Ank^fx^* controls (Fig. 5*U*). Taken together, these data provide evidence that despite the dynamic range of AnkR at the node as it compensates for AnkG loss, AnkR is unable to maintain the nodal complex without AnkG and NF186, and ultimately it begins to destabilize just as is the case for Na_V_ channels and βIV Spectrin.

### Decline in nerve conduction follows timelines of nodal destabilization

**Figure 6.**
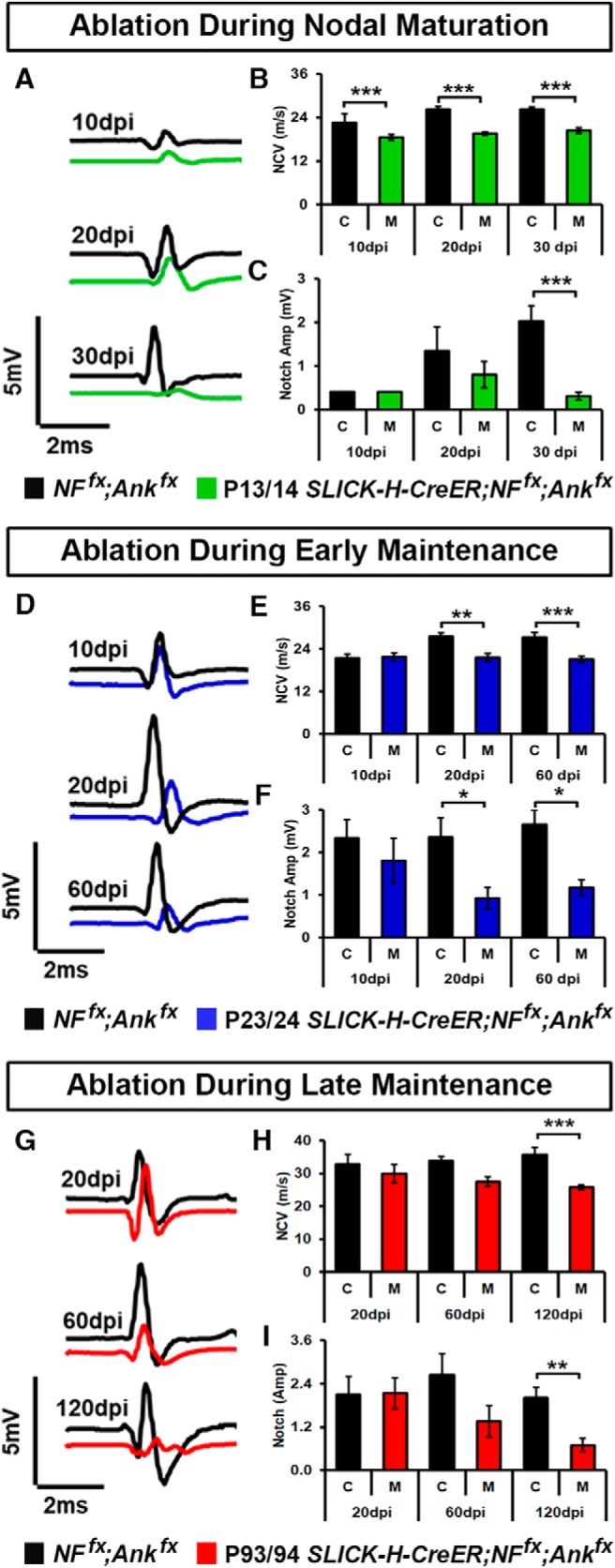
Combined loss of neurofascin 186 and AnkG severely impacts nerve conduction. ***A–C***, Representative electrophysiological profiles (***A***) of compound action potentials (CAPs) from SNs 10, 20, and 30 days after tamoxifen injection during nodal maturation (P13/14) for *NF^fx^;Ank^fx^* (black line) and *SLICK-H-CreER;NF^fx^;Ank^fx^* (green line) with quantification of NCV (***B***) and amplitude (***C***). ***D–F***, Representative electrophysiological profiles (***D***) of CAPs from SNs 10, 20, and 60 days after tamoxifen injection during early nodal maintenance (P23/24) for *NF^fx^;Ank^fx^* (black line) and *SLICK-H-CreER;NF^fx^;Ank^fx^* (blue line) with quantification of NCV (***E***) and amplitude (***F***). ***G–I***, Representative electrophysiological profiles (***G***) of CAPs from SNs of 10, 60, and 120 days after tamoxifen injection during late nodal maintenance (P93/94) for *NF^fx^;Ank^fx^* (black line) and *SLICK-H-CreER;NF^fx^;Ank^fx^* (blue line) with quantification of NCV (***H***) and amplitude (***I***). All data are represented as mean ± SEM (*n* = 6–8 mice/group; two-way ANOVA, Tukey *post hoc* analysis). Black asterisks indicate statistical differences between control and mutant; colored asterisks signify differences between time points among the mutant group.

As changes in the electrophysiological properties of myelinated axons were previously reported after ablation of NF186 or AnkG alone in neurons (Desmazieres et al., 2014; Saifetiarova et al., 2017; Taylor et al., 2017), we next wanted to determine the consequences on nerve conduction of simultaneous ablation of NF186 with AnkG at various time points in nodal development. Thus, we performed *in vivo* electrophysiological recordings from the SN of age-matched controls and double AnkG/NF186 cKOs mice which were ablated at various time points: maturation (P13/14), early maintenance (P23/24), or late maintenance (P93/94; Fig. 6). As early as 10 dpi after P13/14 ablation during nodal maturation, a significant decrease in NCV was already apparent in *SLICK-H;NF^fx;^Ank^fx^* compared to *NF^fx^;Ank^fx^* controls, and this reduced NCV persisted until 30 dpi (Fig. 6*A*, *B*). As is evident from the *NF^fx^;Ank^fx^* controls (black bars), as mice age there is naturally an increase in both the compound action potential (CAP) amplitude (Fig. 6*C*), as well as a more gradual increase in the NCVs (Fig. 6*E*). However, this same increase was not found in the double cKO after P13/14 ablation, and instead a significant decrease was seen in the CAP amplitude compared to age-matched controls (Fig. 6*C*).

While P13/14 ablation led to changes at 10 dpi, no changes in either conduction velocity or CAP amplitude were seen in *SLICK-H;NF^fx;^Ank^fx^* compared to *NF^fx^;Ank^fx^* controls 10 dpi after ablation at P23/24 during early nodal maintenance (Fig. 6*D–F*). By 20 dpi after P23/24 ablation, there was a significant reduction in both NCV and amplitude in double cKO compared to control, which continued at 60 dpi. This is in contrast with both single AnkG and NF186 cKO, which were previously shown to have no reduction in either NCV or amplitude of SN before 4 mpi after ablation at P23/24 (Saifetiarova et al., 2017; Taylor et al., 2017). When AnkG and NF186 were ablated during late nodal maintenance at P93/94, no significant changes in NCV were detected before 60 dpi; although there was a trend toward lower CAP amplitudes at this time point (Fig. 6*G–I*). However, at the terminal time point of 120 dpi after P93/94 ablation, when very little NF186 remains at the nodes, there was a significant reduction in both NCV and CAP amplitude observed in the double cKO compared to age-matched controls. This loss of NCV in the SN after P93/94 ablation of NF186 and AnkG is consistent both with reductions seen 30 dpi after P13/14 and 60 dpi after P23/24 dual ablation and with the decrease seen 6 mpi after P23 ablation of single NF186, but not single ablation of AnkG, which only led to a decrease in amplitude. However, loss of AnkG with NF186 led to changes in NCV at 10 dpi before NF186 levels at the node had dropped. Taken together, these electrophysiological measurements support a synergistic role for NF186 and AnkG in maintaining sufficient levels of Na_V_ channels at nodes to propagate action potentials and sustain proper conduction velocity along myelinated axons. Furthermore, these studies emphasize that delaying the timing of ablation can preserve the integrity of the nodal complex and thereby sustain the electrical properties of myelinated axons for some time. However, NF186 levels falling below critical levels at the node triggers severe nodal degeneration and thus loss of nerve conduction.

### Enhanced destabilization of the nodal complex leads to faster ultrastructural changes in myelinated axons

**Figure 7.**
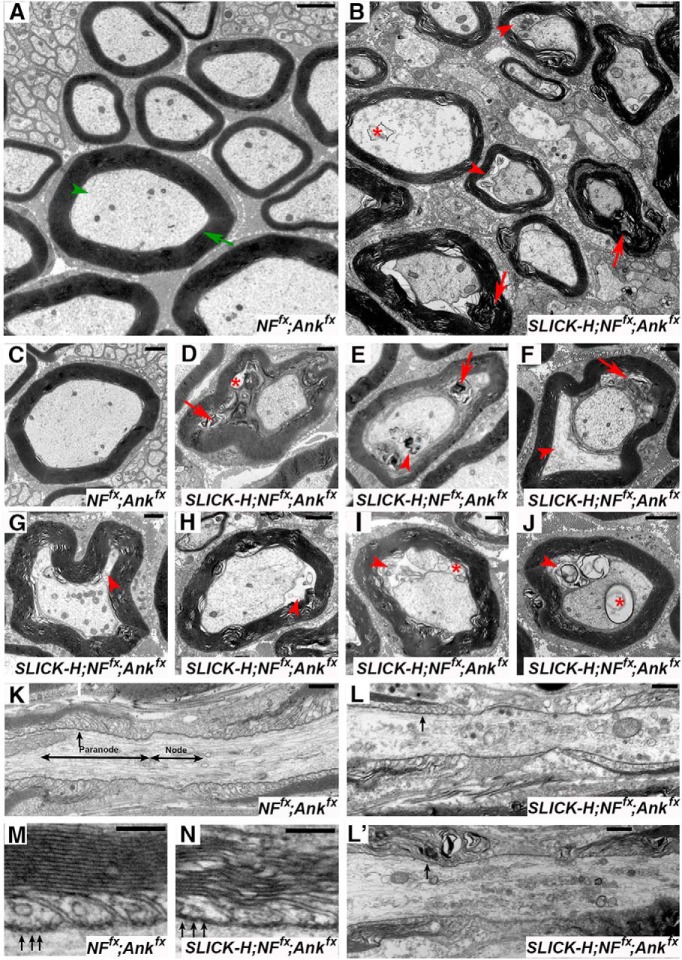
Destabilization of the nodal protein complex leads to sciatic nerve axonal abnormalities. ***A–J***, Transmission electron microscopy (TEM) of cross sections from SNs of *NF^fx^;Ank^fx^* control (***A***, ***C***) and *SLICK-H-CreER;NF^fx^;Ank^fx^* (***B***, ***D–J***) mice 60 days after P23/24 injection. Green arrowheads point to normal axons, red arrowheads to abnormal accumulations in the periaxonal space, green arrows to compact myelin, red arrows to abnormal myelin inclusions, and red asterisks to vacuoles. ***K–N***, TEM of longitudinal sections from SNs of *NF^fx^;Ank^fx^* (***K***, ***M***) and *SLICK-H-CreER;NF^fx^;Ank^fx^* (***L–L′***, ***N***) mice 60 dpi focusing on the nodal and paranodal areas. Black arrows mark paranodal axo-glial septate-like junctions. Scale bar for ***A–J*** = 2 μm, ***K–L′*** = 0.5 μm, ***M***, ***N*** = 0.2 μm.

**Figure 8.**
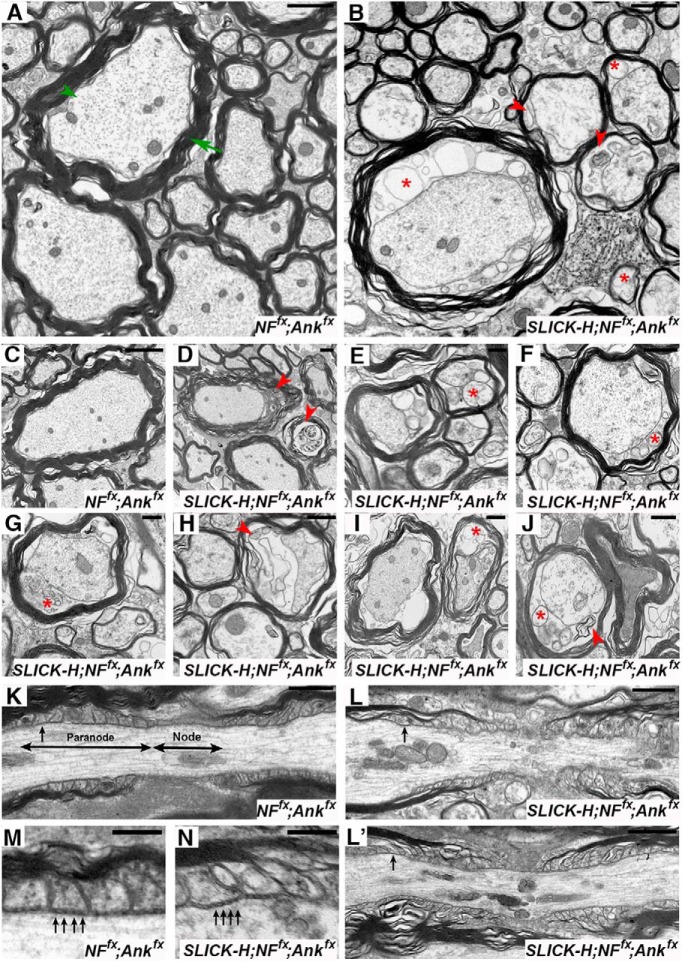
Destabilization of nodes leads to axonopathy in adult CNS myelinated axons. ***A–J***, TEM of cross sections from SCs of *NF^fx^;Ank^fx^* control (***A***, ***C***) and *SLICK-H-CreER;NF^fx^;Ank^fx^* (***B***, ***D–J***) mice 60 days after P23/24 injection. Green arrowheads point to normal axons, greens arrows to compact myelin, red arrowheads to abnormal accumulations in the periaxonal space, and red asterisks to vacuoles. ***K–N***, TEM of longitudinal sections from SCs of *NF^fx^;Ank^fx^* (***K***, ***M***) and *SLICK-H-CreER;NF^fx^;Ank^fx^* (***L–L′***, ***N***) mice 60 dpi focusing on the nodal and paranodal areas. Black arrows mark paranodal axo-glial septate-like junctions. Scale bar for ***A–J*** = 2 μm, ***K–L′*** = 0.5 μm, ***M***, ***N*** = 0.2 μm.

As ablation of NF186 in combination with AnkG led to accelerated nodal disorganization and reduction in nerve conduction compared to single lines, we next inspected if the ultrastructural pathology of myelinated axons was also enhanced in this double ablation model. At 60 dpi after P23/24 ablation of NF186 and AnkG, SNs and SCs from *NF^fx^;Ank^fx^* and *SLICK-H;NF^fx;^Ank^fx^* were processed for electron microscopy under identical conditions; representative images are presented in Figs. 7 and 8, respectively. To closely assess the integrity of the axons, electron micrographs of cross sections through the SN from P23/24 injected controls and the dual cKO were evaluated (Fig. 7*A–J*). The transverse sections from P23/24 injected control SN show morphologically normal axons (green arrowhead) surrounded by compact myelin ensheathment (green arrows, Fig. 7*A*, *C*). While roughly 50% of myelinated axons in the SNs of dual cKOs showed no significant differences in myelin thickness or axon diameter compared to controls, the remaining myelinated fibers from P23/24 ablated *SLICK-H;NF^fx;^Ank^fx^* displayed signs of axonal pathology (Fig. 7*B*, *D–J*). The most frequent axonal morphology observed was axonal atrophy, where the axons are clearly too small for their surrounding myelin sheath (Bilbao and Schmidt, 2014). This axonal shrinking, which is represented in several dual ablated axons in Fig. 7*B*, *D–F*, *J*, was accompanied by abnormalities in the periaxonal region including swellings (red arrowheads), myelin structural alterations (red arrows), or vacuolation (red asterisks). Further, accumulations of debris and disordered presence of organelles was observed in ∼10% of *SLICK-H;NF^fx;^Ank^fx^* axons (Fig. 7*E*, *G*), while vacuolation within the axons was found at a frequency <5% in dual mutant SN but never observed in controls (Fig. 7*B*, J). Electron micrographs of longitudinal sections through the nodal/paranodal regions of SN from P23/24 injected controls and the dual cKO showed preservation of stereotypical axo-glial domains (Fig. 7*K–L′* ). In addition, accumulations of membranous debris and organelles were consistently found at *SLICK-H;NF^fx;^Ank^fx^* nodes (Fig. 7*L–L′* ), which were rarely observed in control fibers (Fig. 7*K*) suggesting axonal cytoskeletal disorganization in the nodal regions. At higher magnification, the paranodal myelin loops from the dual cKO mice (black arrowheads, Fig. 7*N*) showed normal morphology of tightly apposed myelin loops that indent the axons with characteristic electron-dense septa, as was observed in control mice (black arrowheads, Fig. 7*M*) indicating that the uniform arrangement of septa between the myelin loops and the axolemma were maintained in mutant axons in the PNS.

Similar to what was observed in SN cross-sections, ∼1 in 3 axons from dual ablated cKO SCs showed clear signs of axonal pathology (Fig. 8*B*, *D–J*), which was not observed in control SCs (Fig. 8*A*, *C*). As in SN, a significant number of mutant axons in the SCs appeared to have shrunk away from the ensheathing myelin, leaving gaps in periaxonal spaces filled in by vacuoles, organelle accumulations, or uncompacted myelin (Fig. 8*B*, *D–J*). These areas do not represent paranodal regions, which typically show myelin loops still attached to the axons. Preserved nodal and paranodal domains were present in both controls and the dual cKO in SC (Fig. 8*K*, *L′* ). As in SN, accumulations of debris were found to be more dense and frequent in *SLICK-H;NF^fx;^Ank^fx^* than in *NF^fx;^Ank^fx^* age-matched controls. At high magnification of longitudinal sections in SCs, stereotypical electron-dense septa were found between the paranodal loops and the axolemma in both control and mutant animals (Fig. 8*M*, *N*). These data suggest that while the structure of myelin may unravel as axonal shrinkage occurs in SC, it is not accompanied by widespread demyelination in the double cKOs. Overall, the ultrastructural analyses demonstrate that simultaneous ablation of a cytoskeletal component together with a cell adhesion molecule at the nodes has dramatic consequences on myelinated axons, leading to ultrastructural changes associated with axonal pathology. These observations further underscore that AnkG and NF186 work synergistically throughout life to preserve the nodal structure and ultimately to maintain proper axonal architecture for saltatory nerve conduction.

## Discussion

The nodal complex after initial clustering undergoes a period of maturation followed by precise maintenance for the rest of adult life to ensure proper neuronal activity. Perturbations that may affect nodal functions below a certain threshold will dramatically alter neuronal functions. In the current study, we used spatiotemporal tools to address the mutual roles of NF186 and AnkG in the stability of the node and how combined loss of these proteins at different times points during postnatal life impacts nodal stability. Here we report that when NF186 is ablated in combination with AnkG, there is accelerated loss of both these proteins which coincides with enhanced nodal destabilization, significant reduction in nerve conduction, and ultimately quicker demise of myelinated axons compared to ablation of these key nodal proteins individually. Furthermore, we demonstrate that the timing at which ablation occurs determines the rate of nodal destabilization. These studies uncover that nodal stability increases over time during early postnatal development and that maintenance of the steady-state levels of nodal proteins without significant turnover is beneficial for maintaining optimal nerve conduction along myelinated axons.

### Nodal stability and the aging node of Ranvier

**Figure 9.**
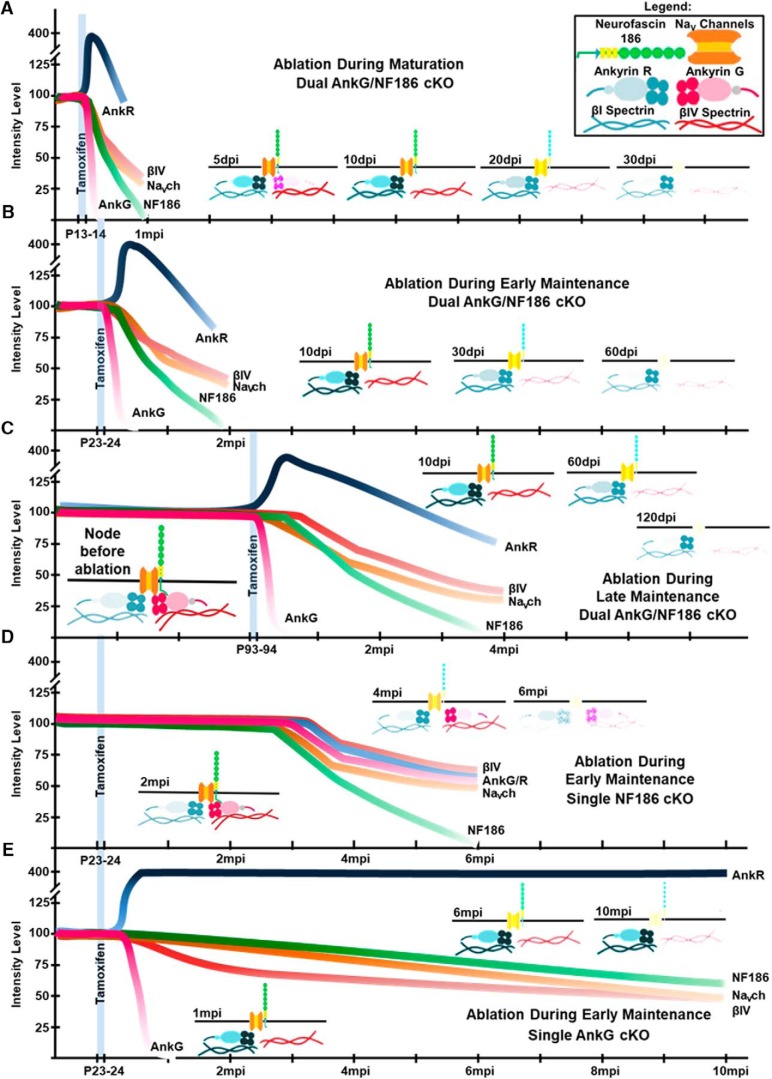
Schematic representation of differential nodal destabilization with age. ***A***, P13/14 ablation of AnkG and NF186 during nodal maturation results in a rapid nodal deterioration. By just 5 dpi, AnkG (pink line) is reduced to below 50%, which leads to a dramatic increase in AnkR (blue line). By 10 dpi, AnkG is completely absent from the node, and βIV Spectrin (red line) is being replaced by the AnkR/βI Spectrin complex. As NF186 (green line) levels significantly fall, corresponding reductions are seen in NaV channels (orange line) followed by βIV Spectrin and AnkR. Mice die ∼1 mpi. ***B***, P23/24 ablation of AnkG and NF186 during early nodal maintenance results in a significantly enhanced timeline of nodal destabilization compared to single cKOs. As with P13/14 ablation, AnkG is completely absent from the node by 10 dpi, and the AnkR/βI Spectrin complex has moved in. However, the half-life of NF186 is increased to 1 mpi with P23/24 dual ablation, and corresponding reductions in Na_V_ channels, βIV Spectrin and AnkR reach a peak at 2 mpi, at which point these mice die. ***C***, P93/94 ablation of AnkG and NF186 during late nodal maintenance results in delayed nodal deterioration compared to dual ablation at earlier time points. Although AnkG is still completely absent from the node by 10 dpi and AnkR has begun to move in, the half-life of NF186 is increased to 2 mpi with dual ablation at 3 months of age. Reductions in Na_V_ channels, βIV Spectrin, and AnkR continue until 4 mpi, when mice die. ***D***, P23/24 single ablation of NF186 during early nodal maintenance results in a gradual reduction of NF186 that is not completely lost until 6 mpi. As NF186 levels significantly decline, corresponding reductions are seen in Na_V_ channels, AnkG, AnkR, and βIV Spectrin. Mice die at 6 mpi. ***E***, P23/24 single ablation of AnkG during early nodal maintenance results in a rapid loss of AnkG and increase of AnkR over the first 1 mpi. However, nodal destabilization occurs very slowly, with βIV Spectrin, Na_V_ channels, and NF186 falling below 50% at 10 mpi, at which point these mice die.

The dual loss of AnkG/NF186 has permitted us to define the timeline and consequences of nodal degeneration *in vivo* at different critical points in nodal stability: maturation and early-to-late maintenance (see Fig. 9). The impact of loss of single components on nodal maintenance revealed that the nodal complex remained remarkably stable for greater than half a year after AnkG or NF186 ablation (Saifetiarova et al., 2017; Taylor et al., 2017). However, it remained unresolved as to what impact loss of these proteins would have on nodes later in adult life. Ablating NF186/AnkG after 3 months (P93) significantly delayed nodal destabilization and furthermore had a profound doubling effect on the lifespan of the mice compared to dual ablated at P23. Interestingly, these late ablated mice did not show a significant reduction in weight compared to their littermate controls, even at the final time point of 120 dpi (Fig. 1), which was seen with both P13 and P23 ablation of NF186 and AnkG. Of note, the increase in lifespan and the delay in nodal destabilization after P93 ablation did not occur because of a difference in the effectiveness of tamoxifen knockdown in older mice, as the number of nodes which showed ablation of AnkG and NF186 were equal across all timelines in both PNS and CNS (Fig. 2). Instead the difference in increased nodal stability was correlated with the increased nodal half-life of AnkG and NF186, so that the core nodal proteins are degraded from the node quickest after ablation at P13 but persisted longer as nodes got older as in P93 or when AnkG or NF186 were ablated individually.

Together, these findings raise the important question of what makes a node different along a myelinated axon at P13, P23, and P93 contributing to its increased stability. While early in development with increasing myelination, nodes of Ranvier are known to undergo a maturation step from Na_V_1.2 to Na_V_ 1.6 (Boiko et al., 2001; Kaplan et al., 2001), the molecular composition of the node has been thought to remain relatively stable between P23 and P93 in SN and SC. Using *in vitro* myelinating coculture studies, a change was shown to occur over time in the way cell adhesion proteins accumulate at the node, which contributes to the slow turnover of these proteins, but this switch in the source of nodal proteins was suggested to happen before P14 in mice (Zhang et al., 2012). Although no changes at the node have been described thus far to account for the increasing stability seen between P23 and P93, one possibility is that, as the node is maintained, perhaps more nodal proteins accumulate; however, we were unable to detect significant changes in either the level or intensities of core nodal components within this time range (data not shown).

Another plausible hypothesis for increased stability is that a difference occurs not in the nodal composition but in the compaction of these proteins at the node with time. In support of this, axonal constriction was described at the node of Ranvier and has been shown to begin before birth and to continue to increase with age until after 8 weeks and then to remain stable into adulthood in feline models (Sward et al., 1995). This nodal constriction has been proposed not only to interfere with axonal transport, limiting the diffusion of proteins from the node, but also to contribute to the age-dependent increase in nerve conduction velocity along myelinated fibers (Halter and Clark, 1993; Sward et al., 1995; Johnson et al., 2015); however, the mechanism for how axonal compaction occurs and whether it is necessary for the increase in nodal stability with age remains to be determined. In addition to understanding how the nodal complex becomes more stable with age, another important question to be addressed is why this increased stabilization evolved. Although age-related declines in myelin have been well described in many animal models (Knox et al., 1989; Albert, 1993; Peters, 2002), the integrity of the node has been shown to remain intact once fully established, while corresponding structural changes to the paranodal region have been reported with age as changes to myelin occur (Hinman et al., 2006).

### Nodal components and their individual and combined roles in nodal maintenance

Although previous studies have addressed the individual roles of the transmembrane component, NF186, or the cytoskeletal component, AnkG, in nodal stability (Desmazieres et al., 2014; Ho et al., 2014; Saifetiarova et al., 2017; Taylor et al., 2017), no other study has reported the combined ablation, allowing for the first direct comparison between the intrinsic stability of these individual proteins and their roles in long-term maintenance of the nodal complex (Fig. 9*B*, *D*, *E*). While after ablation of NF186 at P23, the nodal half-life of NF186 was found to be 3 mpi, when AnkG was ablated with NF186 in adolescence, the nodal half-life of NF186 was significantly reduced to 30 dpi in both PNS and CNS. A corresponding reduction in the half-life of AnkG was also seen in the dual cKO, where AnkG was completely absent from 90% of nodes by 10 dpi compared to the single AnkG cKO, which did not reach the same levels until after 30 dpi. Without both NF186 and AnkG, adequate levels of neither Na_V_ channels nor βIV Spectrin could remain clustered at the node beyond 2 mpi to preserve nodal function, whereas single cKO mice lived beyond 6 mpi. Overall these data provide evidence for a synergistic and interdependent role of AnkG and NF186 in the maintenance of the node, so that the presence of AnkG at the node slows the rate of NF186 loss, while likewise NF186 provides additional stability as a membrane component at the node even when AnkG is lost. However, loss of AnkG makes NF186 vulnerable, leading to its loss from the nodes.

Furthermore, when ablated at P23, the dual NF186/AnkG cKO showed faster kinetics of nodal complex destabilization than the previously studied double NF186/NF155 cKO that had disruption of both the nodes and the flanking paranodal region (Taylor et al., 2017). In these studies, paranodes were shown to contribute to, but not to be required for, the long-term maintenance of the nodal complex; as Na_V_ channels were shown to be preserved at the node throughout the lifespan in P23 ablated single NF155 cKO mice, as well as in other well-characterized paranodal mutant mice (Bhat et al., 2001; Boyle et al., 2001; Pillai et al., 2009; Taylor et al., 2017). The contribution of paranodes to nodal maintenance raises the possibility that the differences in nodal stability seen with age could be due to the presence of intact paranodal domains and their maturation over time. Furthermore, loss of AnkG/NF186 at any time point did not seem to significantly affect the flanking paranodal regions, as observed by immunostaining of paranodal markers (Figs. 2–5) and the ultrastructural analyses of paranodal loops (Figs. 7 and 8). Thus with NF186 and AnkG combined ablation, nodes deteriorate much faster even in the presence of intact paranodes, suggesting intrinsic nodal destabilization. Overall, these current data further support that the intrinsic stability of nodal AnkG with NF186 is both sufficient and essential to the lifelong maintenance of the node.

### Timing of nodal destabilization and its impact on myelinated axons

The sequence of nodal destabilization and the downstream consequences are of particular relevance to many human neuropathies and myelinating disorders including multiple sclerosis (MS), where nodes and paranodes have been shown to undergo progressive disorganization that correlates with disease severity (Craner et al., 2004; Coman et al., 2006; Lonigro and Devaux, 2009; Desmazières et al., 2012; Devaux et al., 2012). While the primary target of MS is the myelin sheath, axonal damage is reported to be a chief factor in the disability of the disease, leading to reduced quality of life (Haines et al., 2011; Luessi et al., 2012). Yet, the underlying cause and timeline of axonal pathology in demyelinating neuropathologies remains an area of active investigation. In our studies, no matter the timing of ablation, all mice that lost AnkG/NF186 showed a similar pattern of nodal destabilization in both PNS and CNS: (1) AnkG was undetectable at the majority of nodes by 10 dpi and AnkR levels increased; (2) NF186 levels were reduced over a span of time which correlated with the lifespan of the mice; and (3) Na_V_ channels, βIV Spectrin, and AnkR diffused from the node as levels of NF186 dropped (Figs. 3–5).

As Na_V_ channel levels were significantly reduced at the node, *in vivo* electrophysiological recordings from the SN showed reduced conduction velocity and amplitude. The first appearance of these decreases in nerve conduction were highly associated with timing of ablation, as loss of NCV was significant early as 10 dpi after early P13/14 ablation but did not appear until after 60 dpi in the late ablation group (Fig. 6). This reduction in nerve conduction was ultimately reflected by paralysis of the mice. As was previously reported after single ablation of AnkG and NF186, in all timelines dual ablation of AnkG/NF186 lead to ultrastructural changes in myelinated axons of both SC and SN consistent with axonal pathology. At a final time point when both NF186 and AnkG were fully lost from the nodes, ultrastructural analysis revealed axonal pathology and signs of abnormal myelin remodeling including infolding loops and budding, which often follows axonal atrophy (Bilbao and Schmidt, 2014); however, no significant level of demyelination was observed (Figs. 7 and 8). Thus, while delaying the ablation of NF186/AnkG slowed the degeneration of the nodal complex, at the final time point all the mice displayed similar motor dysfunction that correlated with changes in axonal structure.

Overall, our data support the idea that nodes of Ranvier continue to mature and achieve greater stability with age, and disrupting nodal integrity by perturbation of one or more proteins either during early maturation or during long-term maintenance leads to a sequential disorganization of the nodal proteins. Our data further highlight that nodal proteins undergo a very slow turnover as nodal compaction increases, which is designed to ensure optimal nerve conduction and axonal health for proper neuronal functions.

## Synthesis

Reviewing Editor: Chay Kuo, Duke University Decisions are customarily a result of the Reviewing Editor and the peer reviewers coming together and discussing their recommendations until a consensus is reached. When revisions are invited, a fact-based synthesis statement explaining their decision and outlining what is needed to prepare a revision will be listed below. The following reviewer(s) agreed to reveal their identity: Jack Rosenbluth.

Reviewer #1

The manuscript from Taylor et al., revised for eNeuro, describes studies that use an inducible Cre/Lox approach to genetically inactivate the node of Ranvier components Ankyrin G and Neurofascin 186 (NF186) simultaneously either in developing or in mature mice. The authors found that nodal complexes disassembled more slowly as the test animals became older, suggesting that nodal complexes become more stable with age. Moreover, nodal complexes in the double mutants dissembled more slowly than in the corresponding single mutants. Overall, the studies appear well done and the mutants have been comprehensively analyzed. The data presented support the conclusions drawn. The authors have responded well to the concerns raised in the review of their Journal of Neuroscience submission. In particular, they have more clearly described the significance of the underlying rationale for the studies. As presented the work provides an increased understanding of how age influences the stability of the node of Ranvier, which is of significance to various demyelinating disorders. I recommend accept as is.

Reviewer #2

Although this study reports findings of interest, the observations on axonal pathology are unconvincing. For example: the myelin 'inclusion' in Fig. 7B could represent an indentation of the axon by the surrounding myelin sheath sectioned so that the connection with the sheath, in another plane, is not apparent. Multiple images of debris between the myelin sheath and the axon do not tell us whether the debris arose from the axon or the sheath or other source. Fig. 7M could represent a section through an abnormal paranode. The authors also give percentages of axons with abnormalities but no supporting numbers or SDs. Figs. 7J and K show axonal inclusions that could represent evidence of axonal pathology, but by and large the axons of the double KO animals do not show clear cut pathological changes. Apparent shrinkage of axons is shown in Figs. 7G-L compared to 7F. But unlike rigid tubes, axons vary somewhat in diameter along their length, and the images shown may therefore not reflect average caliber. In contrast to the cross sections selected, the longitudinal sections in 7B and 7O show no sign of axonal atrophy compared with 7A and 7N. Clear-cut axonal atrophy is also not apparent in the NFCT-stained axons in earlier plates. The sheath abnormalities are unconvincing as well; unraveling of myelin in Figs. 7T-W could represent preparative artifact.

In summary, axonal pathology is a major finding of this study, but the data presented do not support either atrophy or other axonal pathology convincingly. The periaxonal debris reported is of potential interest, but its significance is unclear. This paper as it stands indicates considerable expertise on the part of the authors, and many of the experimental results are worth reporting. Regrettably, the pathology part of the study does not reflect the same level of expertise, and because of defects in that component, the paper in its present form should not be published.
